# Supplementary material for: Haplotype-resolved gapless genome and chromosome segment substitution lines facilitate gene identification in wild rice
Source: Nat Commun. 2024 May 29;15:4573. doi: 10.1038/s41467-024-48845-6 (PMC11137157; doi:10.1038/s41467-024-48845-6)
Supplement: Supplementary file 1 — Supplementary Information [file 41467_2024_48845_MOESM1_ESM.pdf]

Haplotype-resolved gapless genome and chromosome segment substitution lines facilitate gene identification in wild rice

Huang *et al.*

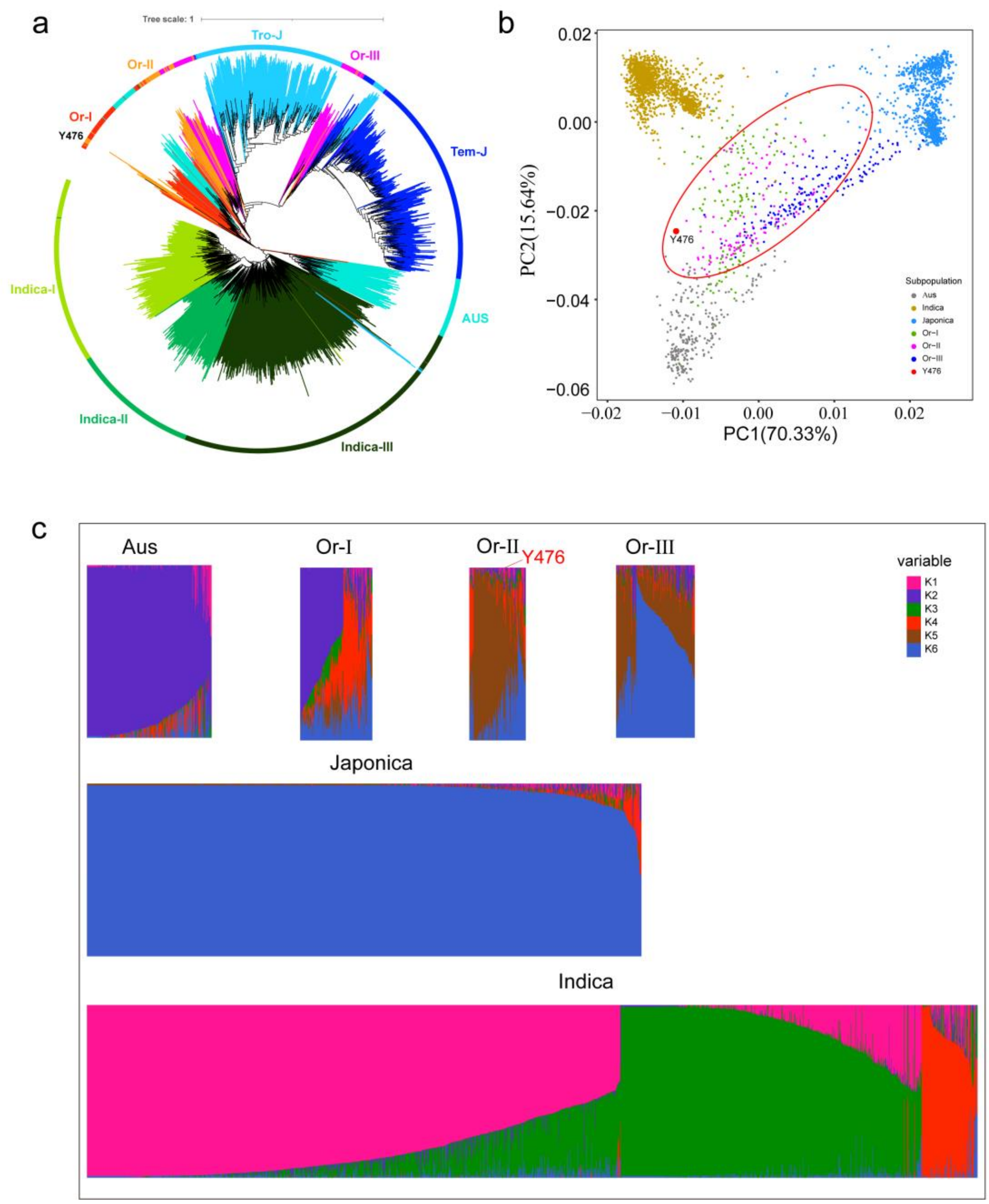

Supplementary Fig. 1. Genetic structure analysis of Y476.

(b) Phylogenetic analysis of wild and cultivated rice species. The neighbor-joining tree was constructed from whole-genome SNPs.

(b) Principal component analysis (PCA) plots of the wild and cultivated rice species. PC1 and PC2 represent the first and second eigenvectors, respectively.

(c) Structure analysis using ADMIXTURE. When K was set to 6, the wild and cultivated rice pool materials were divided into six clusters. The colored bars represent the components corresponding to the subgroup assignments.

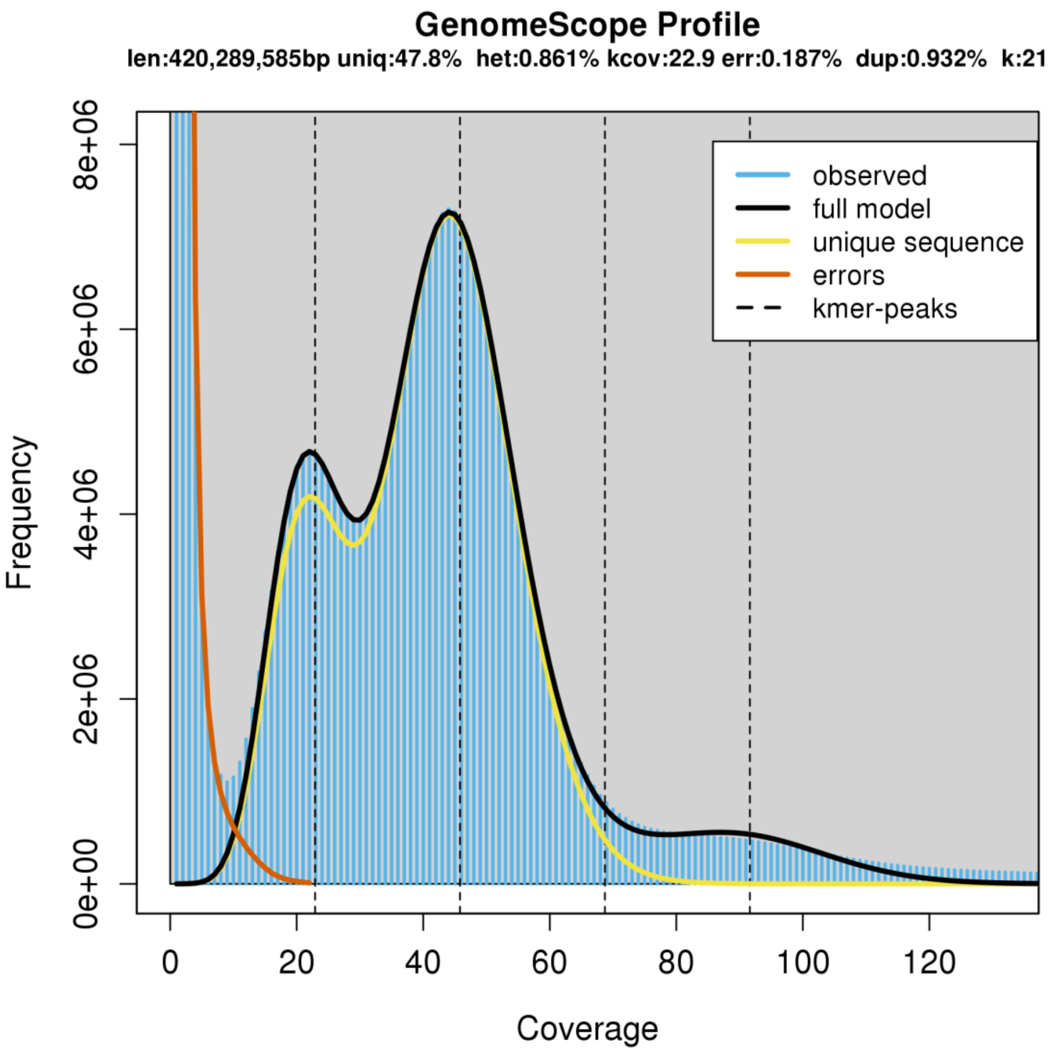

Supplementary Fig. 2. K-mer frequency distribution curve (k-mer = 21) for Illumina short reads of the Y476 genome.

The horizontal axis represents the depth of coverage of the K-mer, and the vertical axis represents the frequency of the depth of the K-mer. The blue line in the figure represents the frequency value. The black line represents the frequency value estimated by the mathematical model. The red line represents the wrong frequency value.

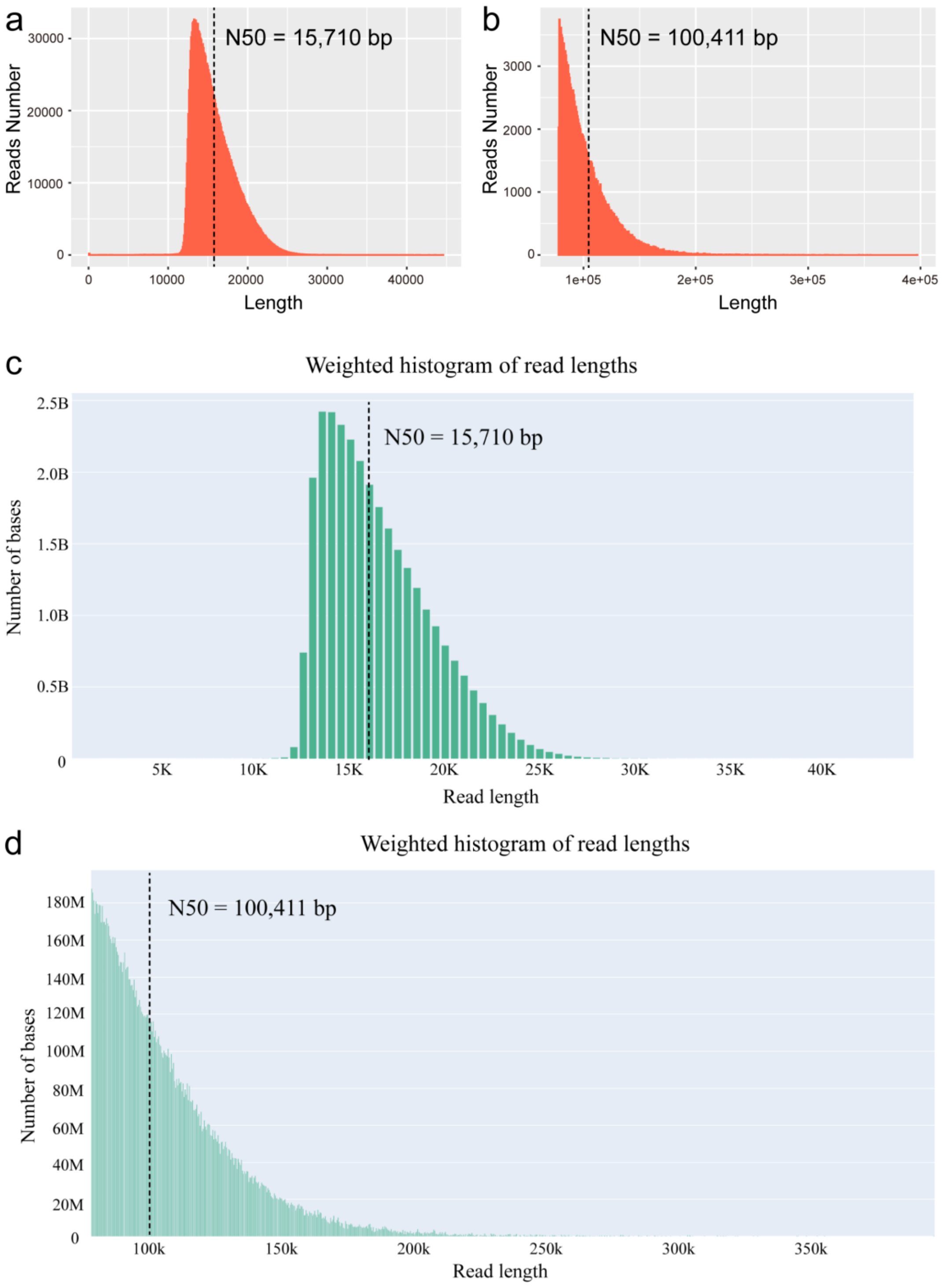

Supplementary Fig. 3. Reads number and total bases distribution with length.

(a) HiFi reads number distribution with length. The X-axis is the length of HiFi reads, and the Y-axis is the number of HiFi reads with the corresponding length.

(b) Ultra-long reads number distribution with length. The X-axis is the length of Ultra-long reads, and the Y-axis is the number of Ultra-long reads with the corresponding length.

(c) HiFi total bases distribution with length. The X-axis is the length of HiFi reads, and the Y-axis is the number of bases with the corresponding length.

(d) Ultra-long total bases distribution with length. The X-axis is the length of Ultra-long reads, and the Y-axis is the number of bases with the corresponding length.

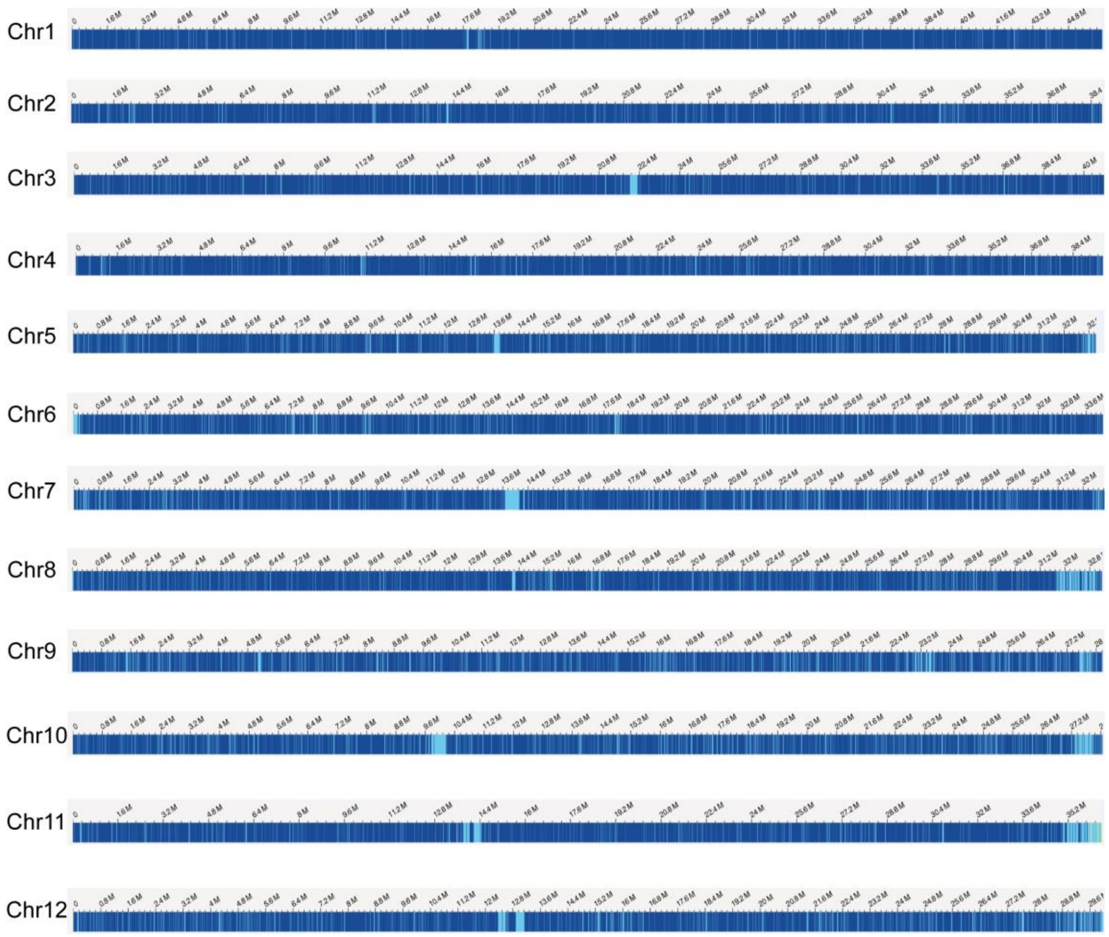

Supplementary Fig. 4. Bionano analysis of the genome assembly.

The blue labels are the aligned labels, whereas the yellow labels are unaligned labels. The panel shows that the Bionano optical mapping results for 12 chromosomes had good quality with no yellow labels.

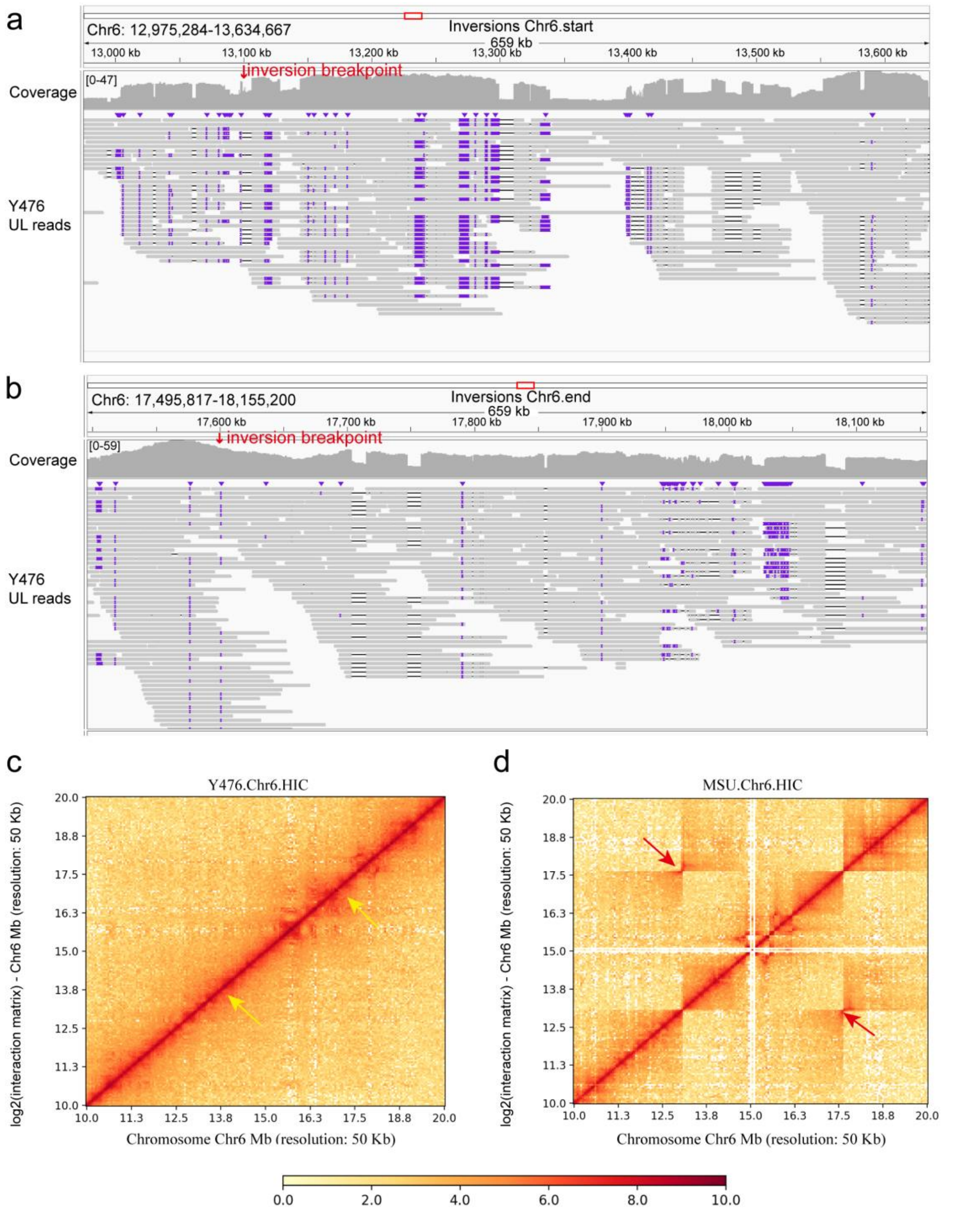

Supplementary Fig. 5. HIC map of a large inversion in Y476 and Nip.

(a) IGV illustration of UL reads (from Y476) mapped to the reference Nip start breakpoints of the inversion. The red arrow represents the inversion breakpoint.

(b) IGV illustration of UL reads (from Y476) mapped to the reference Nip end breakpoints of the inversion. The red arrow represents the inversion breakpoint.

(c-d) Large inversions in Chr6 between Y476 and Nip. The two heatmaps present chromatin interaction matrices mapping the Hi-C data in Y476 against the Nip genome. These maps provide robust support for the large inversion in Chr6.

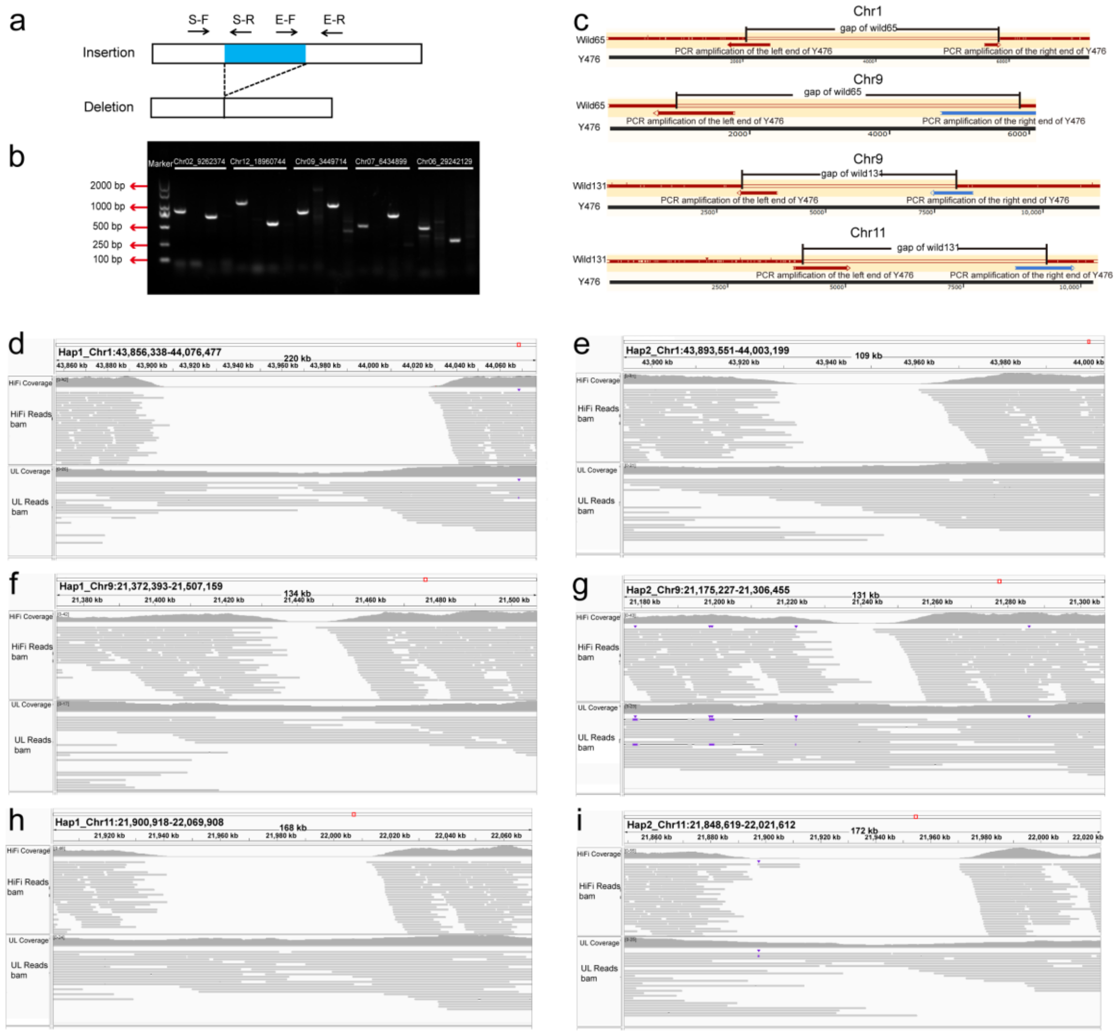

Supplementary Fig. 6. Validation of SVs between the Nip and Y476 genomes by PCR and validation of gaps between Y476 and other wild rice genomes by PCR and HiFi/UL reads.

(a) PCR amplification range during SVs validation.

(b) PCR amplification with the primer pairs in Supplementary Data 7 using DNA samples from Nip and Y476. The order of the samples and primer pairs in each SV is as follows: Nip (S-F/S-R), Y476 (S-F/S-R), Nip (E-F/E-R) and Y476 (E-F/E-R).

(c) PCR validation of representative gaps between the Y476 genome and other wild rice genomes[8]. The black part at the bottom shows a part of the Y476 genome. The red part at the top shows the consistent area between other wild rice genomes and Y476, while the blank part shows the gap compared to Y476. The middle part shows the PCR amplification sequence of the Y476 genome related regions. Red and blue represent the regions where the PCR amplification product sequence aligns with the Y476 genome.

(**d-i**) Above is the coverage depth and read mapping situation of HiFi data at gap upstream and downstream positions; below is the coverage depth and read mapping situation of UL reads. The long reads data were derived from Y476, utilizing Y476 diploids as the reference genome. **d**, **f**, **h** represent the three gap regions of Chr1, Chr9, Chr11 of Hap1, respectively. **e**, **g**, **i** represent the three gap regions of Chr1, Chr9, Chr11 of Hap2, respectively.

Source data are provided as a Source Data file.

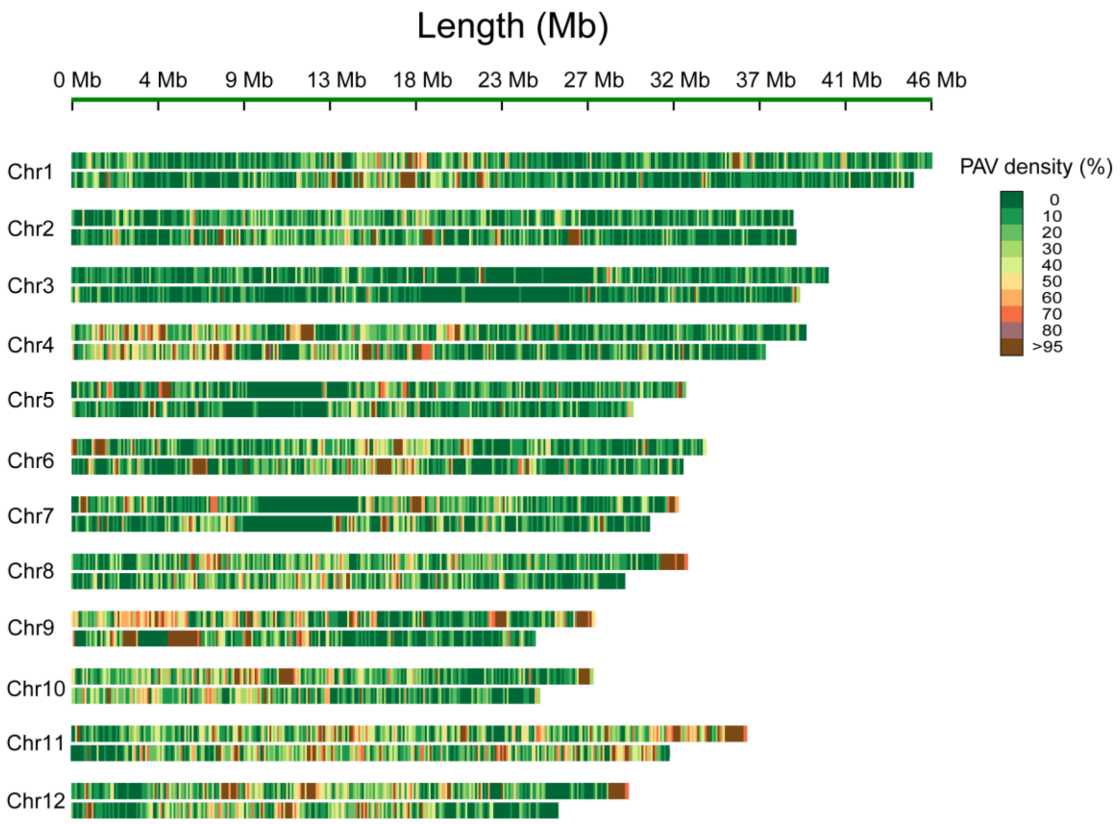

Supplementary Fig. 7. SVs between the Y476 and 9311 genomes.

For each chromosome, the heatmap above shows the SV density in Y476, and the heatmap below shows the SV density in 9311.

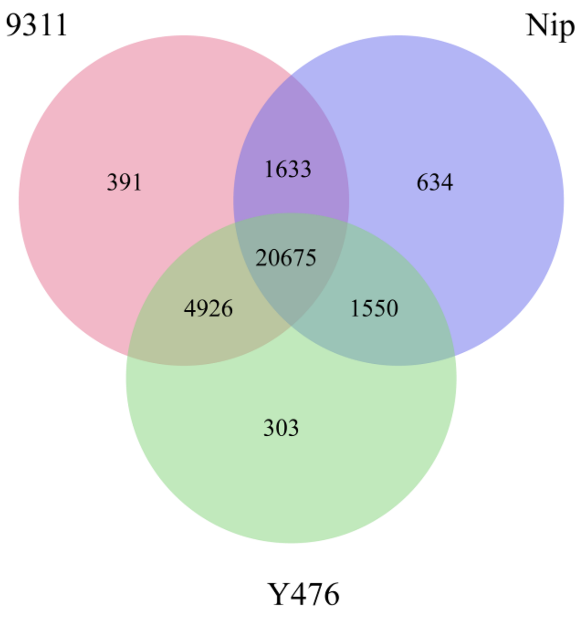

Supplementary Fig. 8. Gene family analysis. Venn diagram showing the shared and unique gene families among Y476, Nip and 9311.

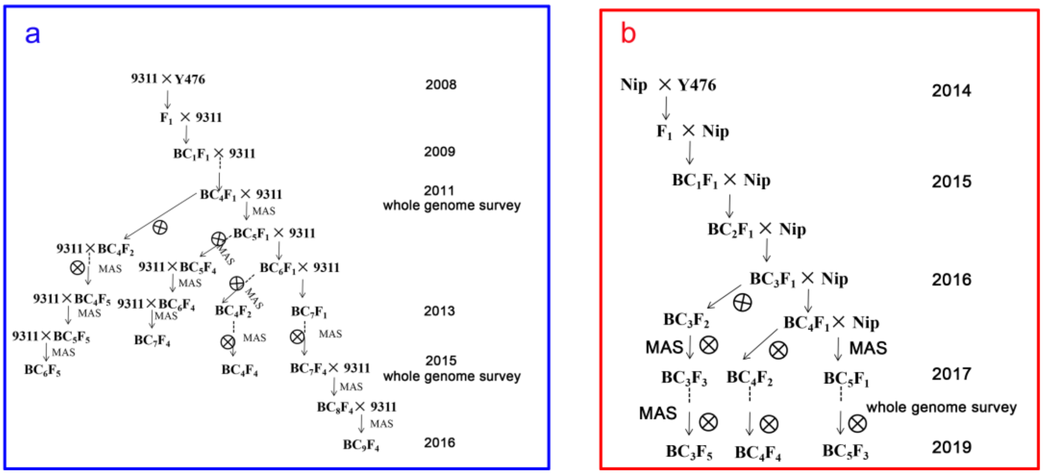

Supplementary Fig. 9. Schematic of the development of chromosome segment substitution lines.

(a) The *O. sativa indica* variety 9311 was used as the recurrent parent, and *O. rufipogon* accession Y476 was used as the donor parent. This CSSL population was constructed over the course of 9 years from 2008 to 2016. The marker assistant selection (MAS) started at the BC_3 generation using SSR and InDel markers. In the BC_4F_1 generation, more than 300 individuals were subjected to the first whole-genome survey. The second whole genome survey was performed at the BC_7 generation to delete the redundant lines. Finally, a set of 198 CSSLs was developed, including 116 single chromosome segment substitution lines (SSSLs).

(b) The *O. sativa japonica* variety Nipponbare was used as the recurrent parent, and Y476 was used as the donor parent. MAS started at the BC_4 generation, and more than 1000 individuals of the BC_4F_1 generation were investigated with MAS. The final population contained 225 CSSLs.

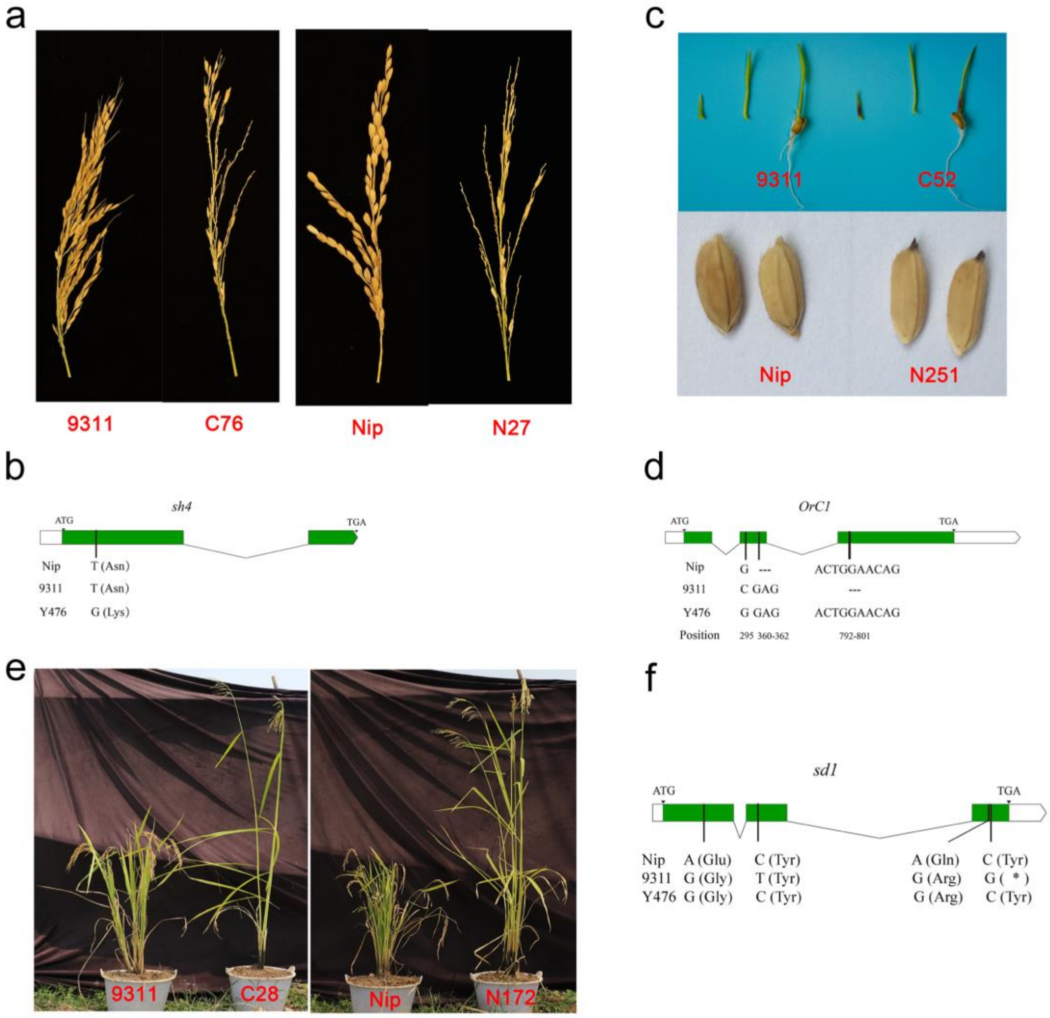

Supplementary Fig. 10. Identification of domestication genes using two sets of CSSL population.

(a) The *sh4* gene is associated with the seed shattering trait. CSSLs C76 and N27 harbor the wild rice *sh4* allele in the 9311 and Nip background, respectively. **(b)** Sequence comparison of the *sh4* gene in Y476, 9311 and Nip, with natural variation, as previously reported[33].

(c) The *C1* gene is associated with purple coloration. CSSLs C52 and N251 harbor the wild rice *C1* allele in the 9311 and Nip background, respectively. **(d)** Sequence comparison of the *C1* gene in Y476, 9311 and Nip, with functional variation, as previously reported[34,35].

(e) The *sd1* gene is associated with plant height. CSSLs C28 and N172 harbor the wild rice *sd1* allele in the 9311 and Nip background, respectively. **(f)** Sequence comparison of the *sd1* gene in Y476, 9311 and Nip, with natural variation, as previously reported[31,32].

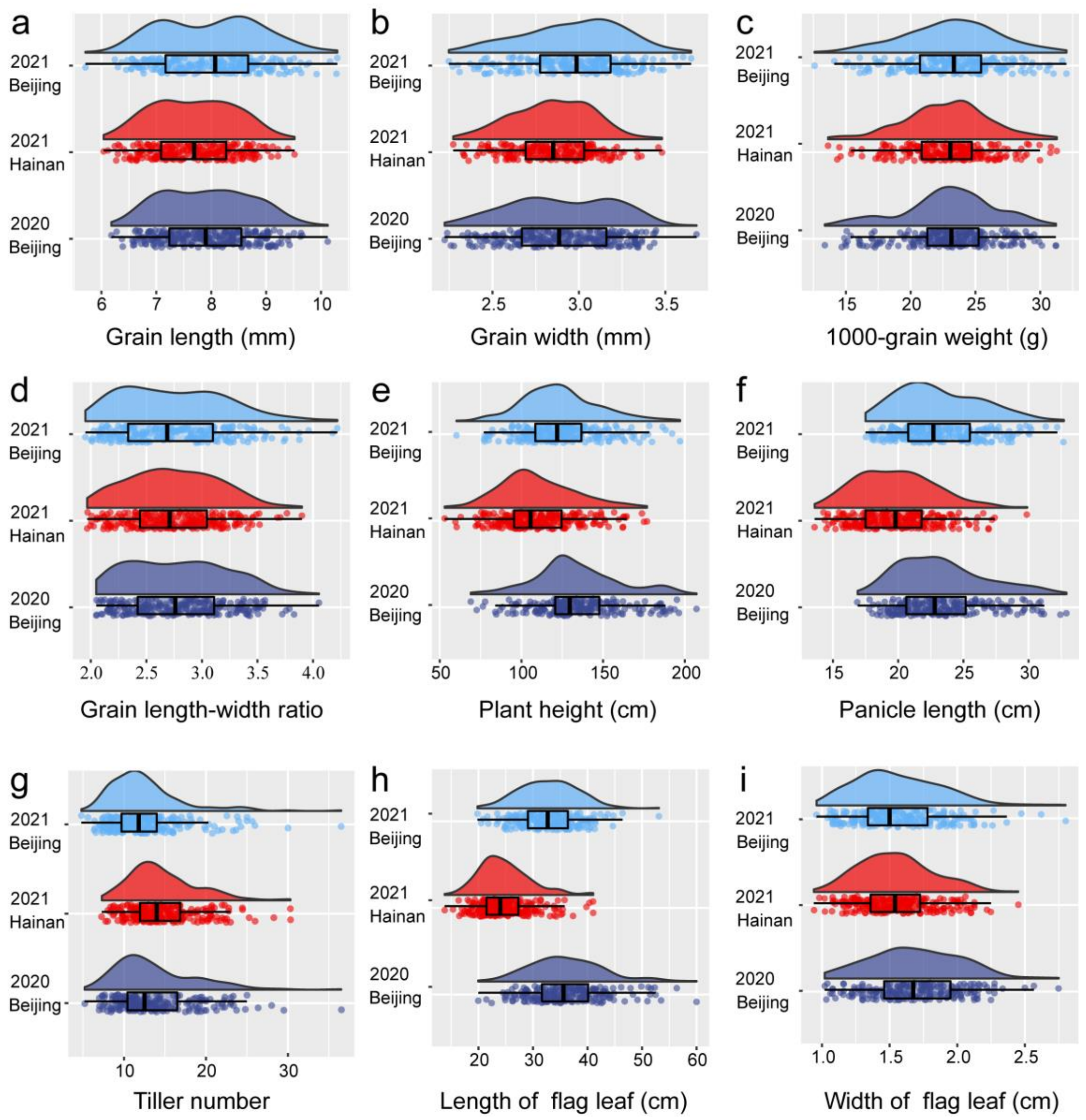

Supplementary Fig. 11. Variability of nine agronomic traits of the CSSL/Nip population in three environments.

(a-i) The variation range of grain length (**a**), grain width (**b**), 1000-grain weight (**c**), grain length-width ration (**d**), plant height (**e**), panicle length (**f**), tiller number (**g**), length of flag leaf (**h**), width of flag leaf (**i)** in three environments. The characteristics of each environment are listed in Supplemental Table 17. Source data are provided as a Source Data file.

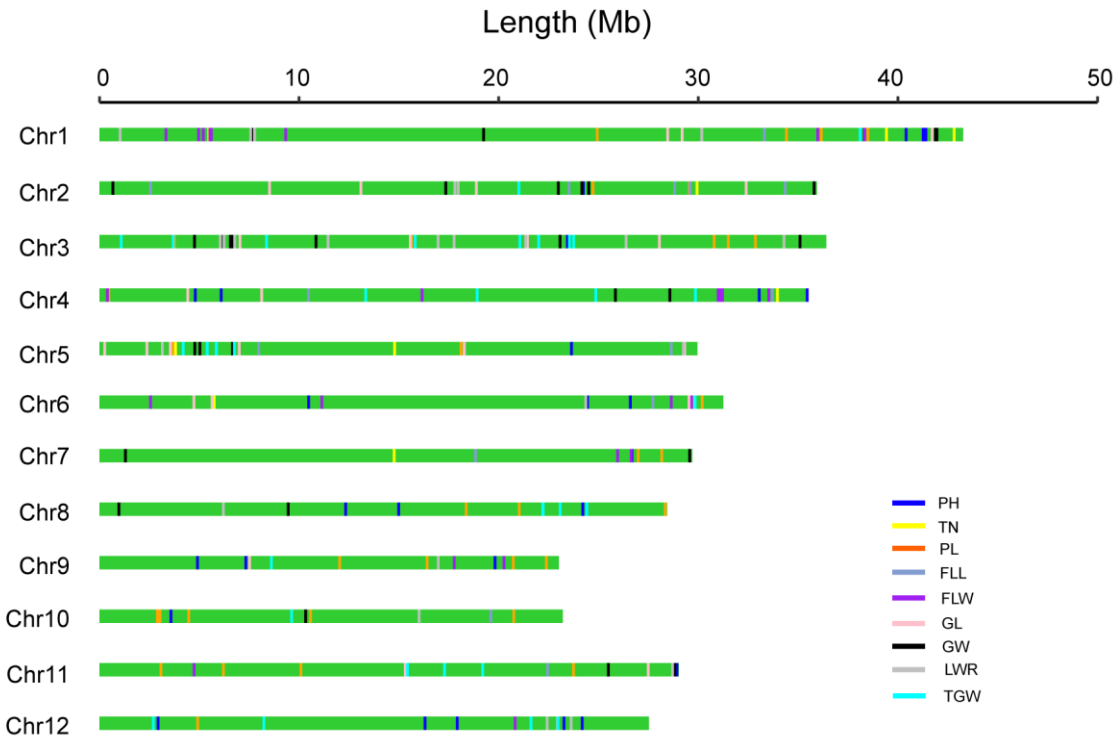

Supplementary Fig. 12. Distribution of agronomic trait-related QTLs in chromosomes. Red, yellow, orange, green, purple, pink, black, grey, and cyan correspond to QTLs related to plant height (PH), number of tills (TN), panicle length (PL), length of flag leaf (FLL), width of flag leaf (FLW), grain length (GL), grain width (GW), grain length-width ratio (LWR) and 1000-grain weight (TGW), respectively. Source data are provided as a Source Data file.

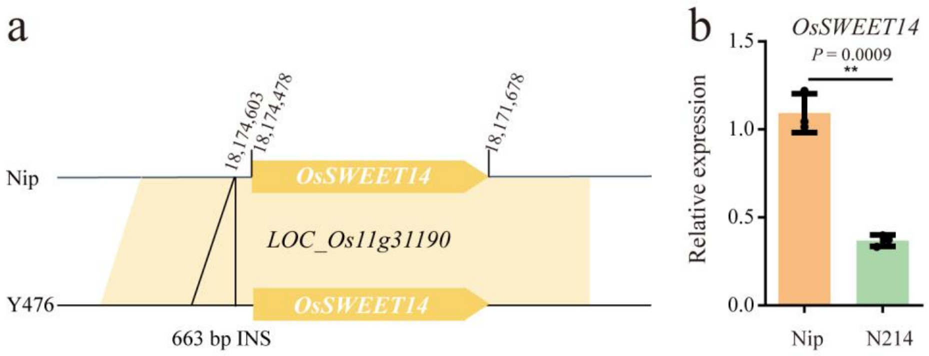

Supplementary Fig. 13. Natural variation and expression levels of *OsSWEET14* between Nip and Y476/N214.

(a) Natural variation of the *OsSWEET14* gene between Y476 and Nip. A 663 bp insertion was found upstream of this gene.

(b) Expression levels of *OsSWEET14* in young panicles of N214 and Nip. CSSL N214 harbors the Y476 allele in the Nip genetic background. Results are presented as the mean ± SD from three biological replicates (n = 3). Comparisons were performed by two-tailed Student's *t*-test (*P < 0.05, **P < 0.01).

Source data are provided as a Source Data file.

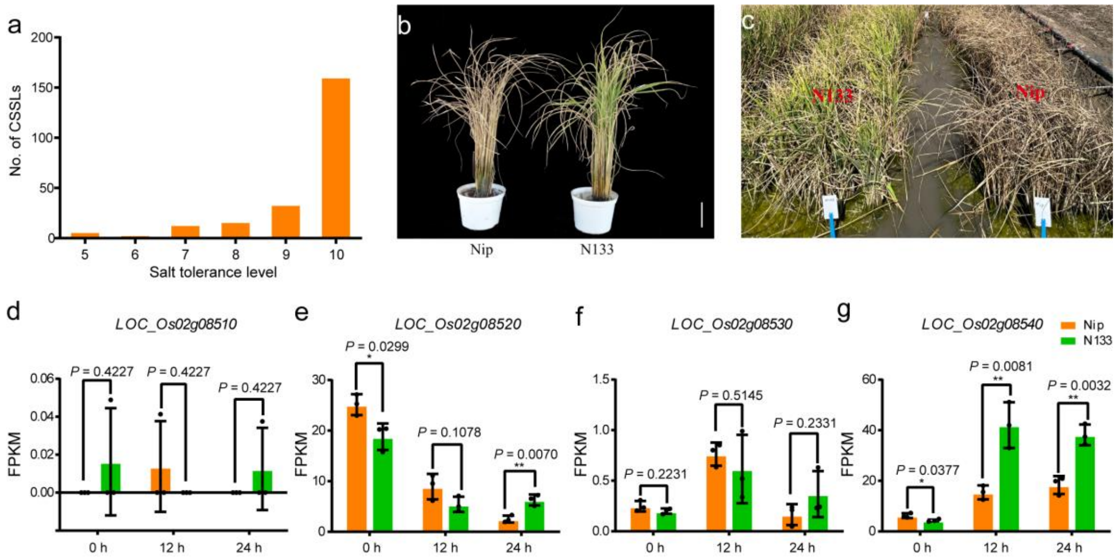

Supplementary Fig. 14. Salt tolerance gene identification and germplasm innovation.

(a) Salt tolerance levels in the CSSL/Nip population. The 225 CSSLs were planted under 85 mM NaCl (0.5% salt stress) during the entire growth period. Salt tolerance levels were defined as 1~10 (high salt tolerance to low salt tolerance) according to established evaluation criteria for rice germplasm salt tolerance[75]. The salt tolerance level of Nip was 10. Note: level 10 represents plant death.

(b) Phenotypes of N133 and Nip grown under 85 mM NaCl during the entire growth period. Scale bar = 10 cm.

(c) N133 showed significantly enhanced salt tolerance under 85 mM NaCl in a paddy field.

(d-g) Expression patterns of four genes in the *S2_4579633* locus of N133 and Nip under salt stress. Results are presented as the mean ± SD from three biological replicates (n =3). Comparisons were performed by two-tailed Student's *t*-test (*$P < 0.05$, **$P < 0.01$).

Source data are provided as a Source Data file.

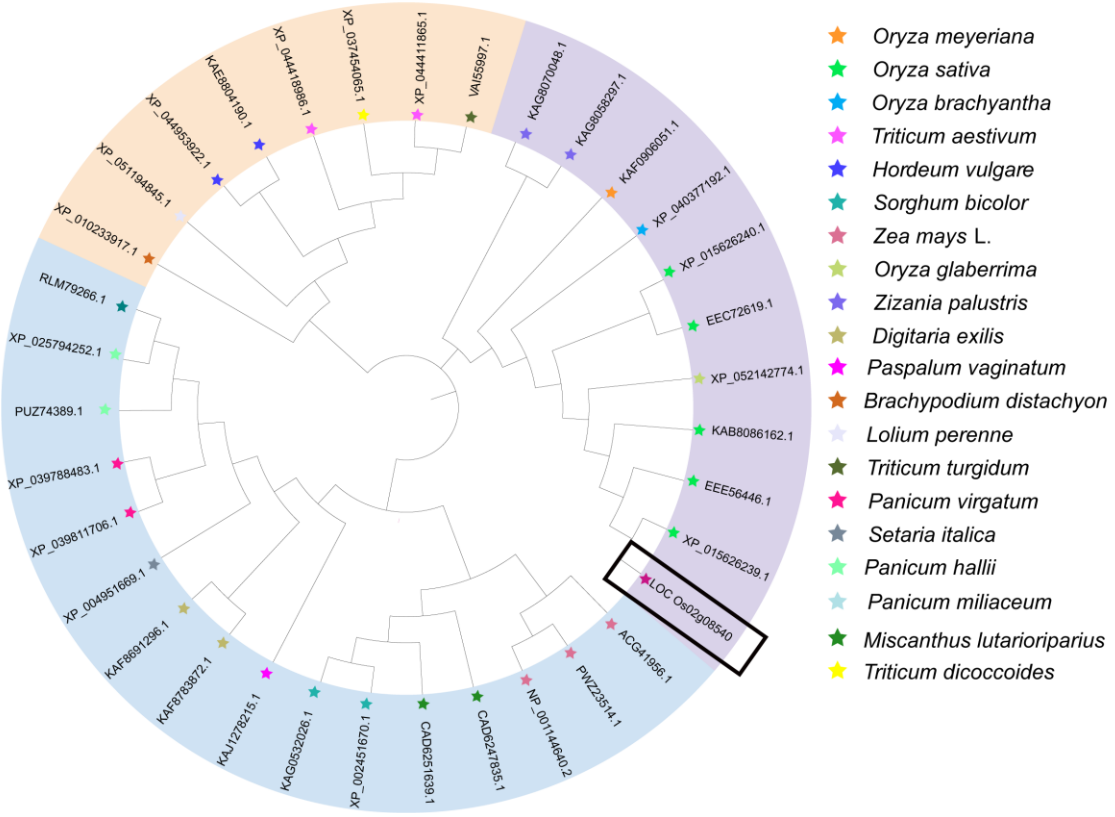

Supplementary Fig. 15. Phylogenetic analysis of *LOC_Os02g08540*.

Phylogenetic analysis of *LOC_Os02g08540* homologs in cultivated rice, wild rice, wheat, barley, sorghum, maize and other plants. *LOC_Os02g08540* is mainly clustered with homologous genes in cultivated rice, and there are also homologous genes in *Oryza glaberrima* and *Oryza brachyantha*. The phylogenetic tree was generated by MEGA 7.0 using the neighbor-joining method.

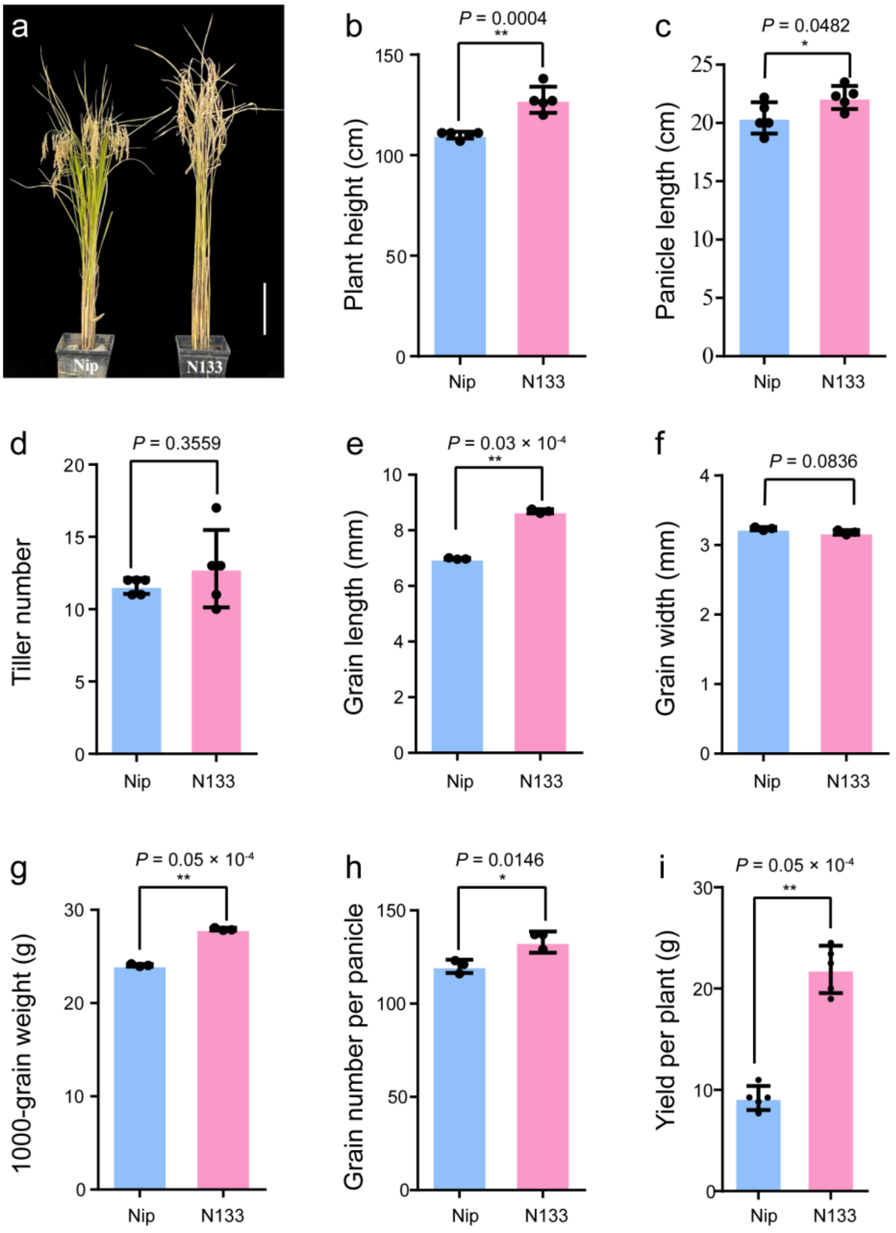

Supplementary Fig. 16. Yield-related traits of the N133 line and the recurrent parent Nip under normal conditions.

(a) Comparison of the plant architecture of Nip and N133. Scale bar = 20 cm.

(b-d) Statistical analysis of plant height (**b**), panicle length (**c**) and tiller number (**d**). Results are presented as the mean ± SD from five biological replicates (n = 5). Comparisons were performed by two-tailed Student's *t*-test (*$P < 0.05$, **$P < 0.01$).

(e-h) Statistical analysis of grain length (**e**), grain width (**f**), 1000-grain weight **(g),** grain number per panicle (**h**). Results are presented as the mean ± SD from three biological replicates (n = 3). Comparisons were performed by two-tailed Student's *t*-test (*$P < 0.05$, **$P < 0.01$).

(i) Statistical analysis of yield per plant. Results are presented as the mean ± SD from three biological replicates (n = 5). Comparisons were performed by two-tailed Student's *t*-test (*$P < 0.05$, **$P < 0.01$).

Source data are provided as a Source Data file.

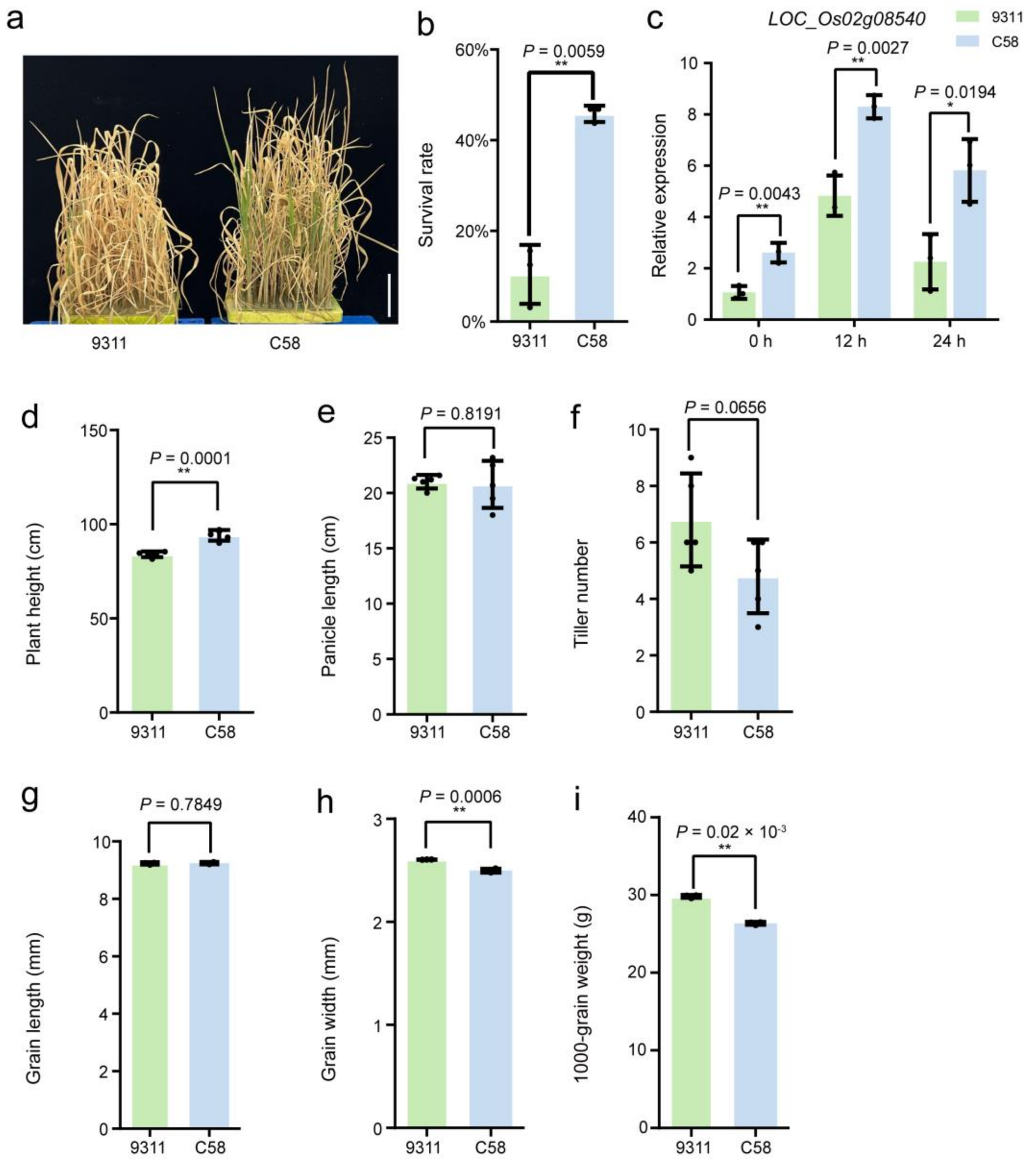

Supplementary Fig. 17. C58, the near-isogenic line of *LOC_Os02g08540*, had significantly higher salt tolerance than background parent 9311.

(a-b) Twenty-day-old seedlings of 9311 and C58 were treated with 150 mM NaCl for six days and recovered in fresh water for six days. Scale bar = 5 cm. (**a**), Survival rate (**b**). Results are presented as the mean ± SD from three biological replicates (n =3). Comparisons were performed by two-tailed Student' s *t*-test (*P < 0.05, **P < 0.01).

(c) Expression levels of *LOC_Os02g08540* in 9311 and C58 at 0 h, 12 h and 24 h after treatment with 150 mM NaCl. Results are presented as the mean ± SD from three biological replicates (n =3). Comparisons were performed by two-tailed Student's *t*-test (**P* < 0.05, ***P* < 0.01).

(d-f) Statistical analysis of the plant height (**d**), panicle length (**e**) and tiller number (**f**) of C58 and 9311. Results are presented as the mean ± SD from five biological replicates (n = 5). Comparisons were performed by two-tailed Student's *t*-test (**P* < 0.05, ***P* < 0.01).

(g-i) Statistical analysis of the grain length (**g**), grain width (**h**) and 1000-grain weight (**i**) of C58 and 9311. Results are presented as the mean ± SD from three biological replicates (n = 3). Comparisons were performed by two-tailed Student's *t*-test (**P* < 0.05, ***P* < 0.01).

Source data are provided as a Source Data file.

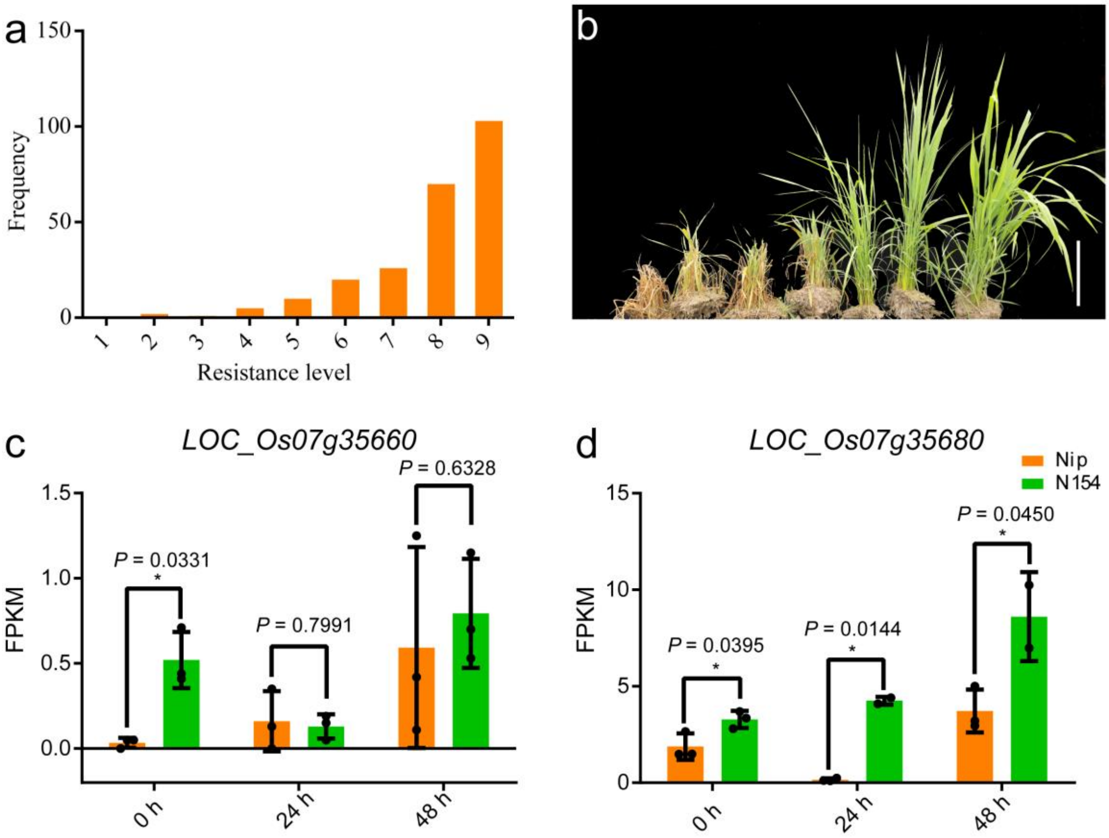

Supplementary Fig. 18. Phenotypes of CSSLs/Nip after inoculation with *M. oryzae* and expression levels of candidate genes.

(a) Rice blast resistance levels in the CSSL/Nip population. The 225 CSSLs were planted in a paddy field and infected with rice blast isolates "FJ07-5-2" and "FJ07-8-1" at 45 days after germination. Rice blast resistance levels were defined as 1~9 (from high to low) according to established evaluation criteria for rice germplasm[75]. The rice blast resistance level of Nip was 9.

(b) Phenotypes of different blast resistance levels in a paddy field after inoculation with *M. oryzae* at the seedling stage. Levels from right to left: 9, 8, 7, 6, 5, 4, and 3. Scale bar = 10 cm.

(c,d) Expression pattern of *LOC_Os07g35660*, *LOC_Os07g35670*, and *LOC_Os07g35680* in the *S2_4579633* locus of N154 and Nip. Expression of *LOC_Os07g35670* was not detected in the transcriptomic data. Results are presented as the mean ± SD from three biological replicates (n = 3). Comparisons were performed by two-tailed Student's *t*-test (*$P < 0.05$, **$P < 0.01$).

Source data are provided as a Source Data file.

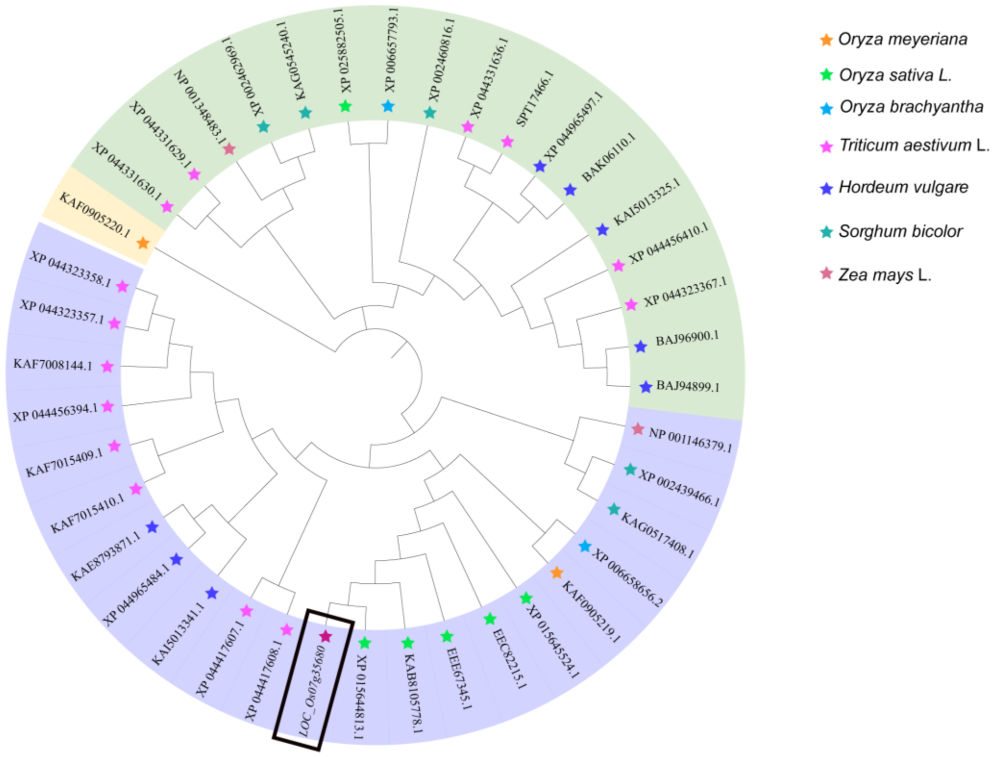

Supplementary Fig. 19. Phylogenetic analysis of *LOC_Os07g35680*.

Homologous genes in *Oryza meyeriana* and *Oryza brachyantha* are clustered closely to *LOC_Os07g35680*. The phylogenetic tree was generated by MEGA 7.0 using the neighbor-joining method.

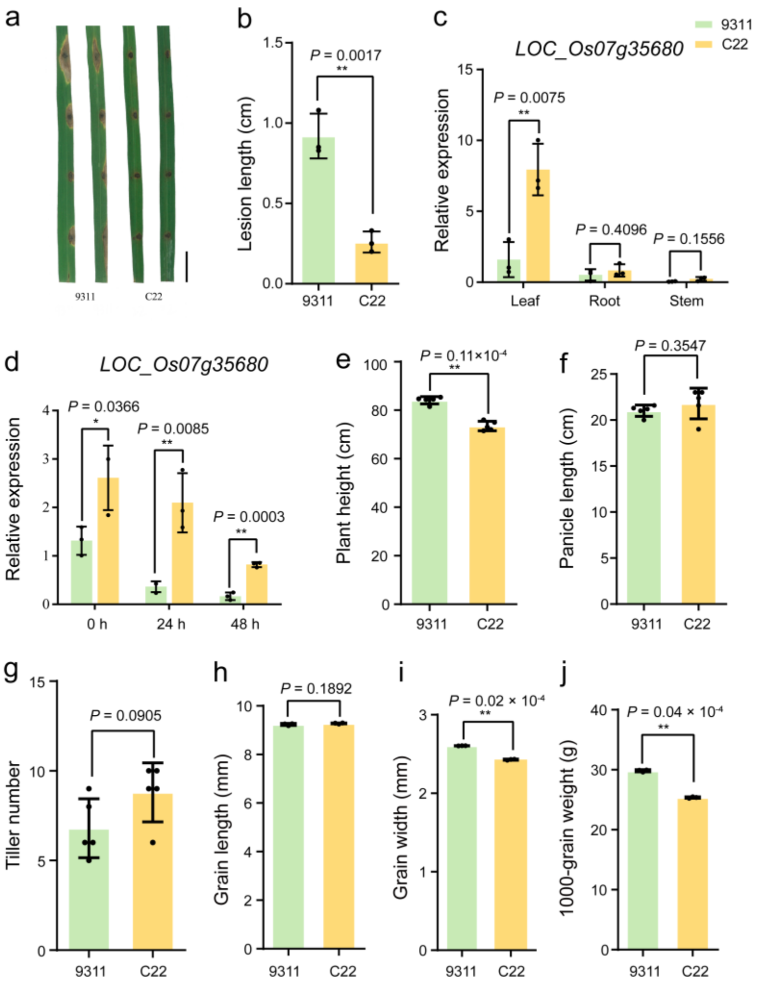

Supplementary Fig. 20. The rice blast resistance of C22, a near-isogenic line of *LOC_Os07g35680*, was significantly higher than that of 9311.

(a) Disease symptoms of 9311 and C22 after inoculation with *M. oryzae*. Scale bar = 1 cm.

(b) Lesion length of 9311 and C22 after inoculation with *M. oryzae*. Lesion area was measured at 7 dpi. Results are presented as the mean ± SD from three biological replicates (n = 3). Comparisons were performed by two-tailed Student's *t*-test (*$P < 0.05$, **$P < 0.01$).

(c) Expression patterns of *LOC_Os07g35680* in different tissues of 9311 and C22. Results are presented as the mean ± SD from three biological replicates (n = 3). Comparisons were performed by two-tailed Student's *t*-test (*$P < 0.05$, **$P < 0.01$).

(d) Expression levels of *LOC_Os07g35680* in 9311 and C22 at 0 h, 24 h and 48 h after inoculation with *M. oryzae*. Results are presented as the mean ± SD from three biological replicates (n = 3). Comparisons were performed by two-tailed Student's *t*-test (*$P < 0.05$, **$P < 0.01$).

(e-g) Statistical analysis of plant height (**e**), panicle length (**f**) and tiller number (**g**). Results are presented as the mean ± SD from five biological replicates (n = 5). Comparisons were performed by two-tailed Student's *t*-test (*$P < 0.05$, **$P < 0.01$).

(h-j) Statistical analysis of grain length (**h**), grain width (**i**) and 1000-grain weight (**j**). Results are presented as the mean ± SD from three biological replicates (n = 3). Comparisons were performed by two-tailed Student's *t*-test (*$P < 0.05$, **$P < 0.01$).

Source data are provided as a Source Data file.

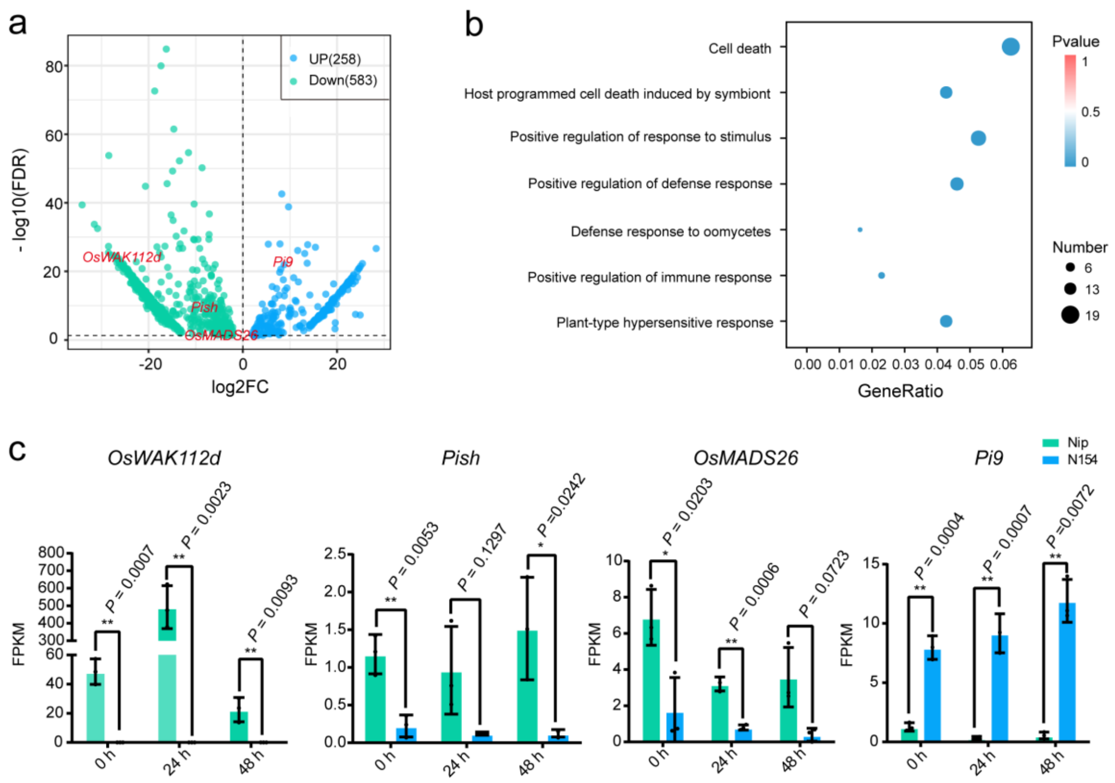

Supplementary Fig. 21. Transcriptome analysis of N154 and Nip.

(a) Volcano plot of DEGs in Nip and N154. Each dot represents a gene, and four documented rice blast resistance genes (*Pi9*, *OsWAK112d*, *OsMADS26* and *Pish*) are labeled in red.

(b) Gene Ontology (GO) terms enriched in the DEGs for Nip and N154. Two-week-old rice leaves were used for transcriptome assays, and the 841 DEGs that overlapped were subjected to GO analysis.

(c) Expression levels of four rice blast-related genes according to the transcriptomic data. Results are presented as the mean ± SD from three biological replicates (n = 3). Comparisons were performed by two-tailed Student's *t*-test (*$P < 0.05$, **$P < 0.01$).

Source data are provided as a Source Data file.

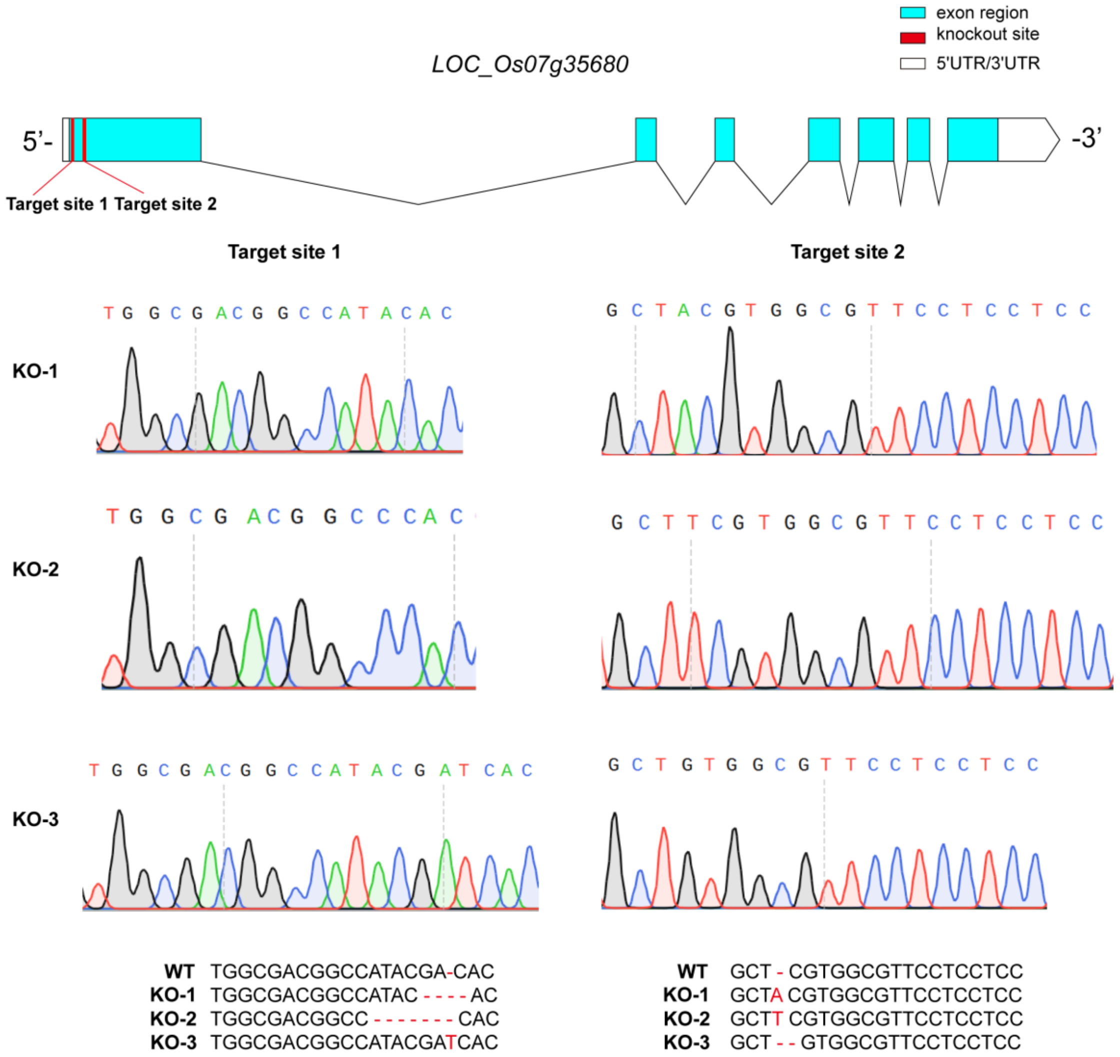

Supplementary Fig. 22. Mutation sites in the *LOC_Os07g35680* gene in N154 line knockout mutants generated by CRISPR/Cas9 editing.

Supplementary Table 1. Summary of the sequence data for Y476.

Sequencing method	Raw data (bp)	Number of subreads	Mean subread length (bp)	Depth (X)	Tissue
HiFi	29,688,336,424	1,875,461	15,829	70.9	
Nanopore	16,703,394,310	163,201	102,348	39.9	
Illumina	26,224,111,800	174,827,412	150	62.6	
HiC	68,900,773,800	459,338,492	150	164.5	
RNA-seq	8,208,116,100	54,720,774	150	\	leaf
RNA-seq	9,136,626,000	60,910,840	150	\	root
RNA-seq	8,492,896,500	56,619,310	150	\	stem
RNA-seq	8,606,405,100	57,376,034	150	\	leaf
RNA-seq	7,877,219,100	52,514,794	150	\	stem
RNA-seq	9,249,345,000	61,662,300	150	\	young panicle
BioNano	972,034,161,542	4,361,201	223,000	2,321	

Supplementary Table 2. Statistics of repeat content in Y476.

Genome name	Hap1			Hap2			Primary		
Chromosome number	12			12			12		
Total length (bp)	411,067,696			411,866,958			418,777,094		
GC percentage	44.15%			44.13%			44.16%		
bases masked (bp)	239,710,859 (58.31%)			240,727,453 (58.45%)			247,220,022 (59.03%)		
	Number of elements	Length occupied (bp)	Percentage of sequence (%)	Number of elements	Length occupied (bp)	Percentage of sequence (%)	Number of elements	Length occupied (bp)	Percentage of sequence (%)
Class I: Retroelements	82,259	124,352,533	30.25	82,781	123,569,014	30.00	83,570	127,144,982	30.36
SINEs	9,821	1,531,876	0.37	9,838	1,541,959	0.37	9,875	1,541,870	0.37
LINEs	16,965	5,707,485	1.39	17,122	5,754,568	1.40	17,204	5,812,654	1.39
LTR elements：	55,473	117,113,172	28.49	55,821	116,272,487	28.23	56,491	119,790,458	28.60
Ty1/Copia	9,766	13,134,879	3.20	9,866	13,366,934	3.25	9,841	13,347,777	3.19
Gypsy/DIRS1	39,521	102,216,572	24.87	40,216	101,441,863	24.63	40,482	104,700,900	25.00
Class II: DNA Transposon	407,014	90,805,984	22.09	406,814	91,107,277	22.12	409,614	91,944,807	21.96
Unclassified	57,811	11,420,647	2.78	58,208	12,239,648	2.97	58,773	12,729,890	3.04

Supplementary Table 3. Statistics of gene annotation of Y476 genome.

Genes annotation statistics	Hap1	Hap2	Primary
Number of genes	36,150	36,336	36,422
Mean gene length (bp)	2,796	2,793	2,784
Mean CDS length (bp)	1,175	1,173	1,171
Mean exons per gene	4.57	4.55	4.55

Supplementary Table 4. Statistics of mapping rate and coverage rate for three data types in Y476 genome.

Sequencing method	Hap1		Hap2		Primary	
	mapping rate (%)	coverage rate (%)	mapping rate (%)	coverage rate (%)	mapping rate (%)	coverage rate (%)
ONT	99.97	99.99	99.97	99.99	99.97	100.00
HiFi	99.87	100.00	99.90	100.00	99.87	100.00
WGS	99.13	99.97	99.14	99.96	99.16	99.97

Supplementary Table 5. The percentage of coverage depth for different ranges of HiFi, ONT and WGS.

Sequencing method	Hap1		Hap2		Primary	
	Coverage depth (X)	Percentage (%)	Coverage depth (X)	Percentage (%)	Coverage depth (X)	Percentage (%)
HiFi	<16	1.74	<16	2.12	< 28	0.68
	16-32	56.04	16-32	55.77	28-48	6.26
	32-48	41.87	32-48	41.59	48-68	46.73
	>48	0.34	>48	0.51	68-88	43.98
					88-108	1.86
					> 108	0.48
ONT	<6	0.98	<6	0.86	< 11	0.06
	6-16	50.65	6-16	50.01	11-21	1.98
	16-26	47.15	16-26	47.98	21-31	11.30
	>26	1.22	>26	1.15	31-41	44.89
					41-51	37.56
					51-61	3.77
					> 61	0.43
WGS	<10	2.96	<10	3.64	< 20	0.05
	10-24	61.28	10-24	59.05	20-40	3.99
	24-38	35.52	24-38	36.23	40-60	59.88
	>38	0.25	>38	1.08	60-80	34.89
					80-100	0.43
					> 100	0.76

Supplementary Table 6. BUSCOs analysis of Y476 genome completeness.

Description	Hap1		Hap2		Primary	
	Number	Percentage (%)	Number	Percentage (%)	Number	Percentage (%)
Complete BUSCOs (C)	1,593	98.7	1,589	98.5	1,594	98.8
Complete and single-copy BUSCOs (S)	1,559	96.6	1,556	96.4	1,559	96.6
Complete and duplicated BUSCOs (D)	34	2.1	33	2.0	35	2.2
Fragmented BUSCOs (F)	14	0.9	16	1.0	13	0.8
Missing BUSCOs (M)	7	0.4	9	0.6	7	0.4
Total BUSCO groups searched	1,614	100.0	1,614	100.0	1,614	100.0

Supplementary Table 7. Protein BUSCOs analysis of Y476 predicted genes.

Description	Hap1		Hap2		Primary	
	Number	Percentage (%)	Number	Percentage (%)	Number	Percentage (%)
Complete BUSCOs (C)	1,565	97.0	1,567	97.1	1,581	98.0
Complete and single-copy BUSCOs (S)	1,534	95.0	1,542	95.5	1,552	96.2
Complete and duplicated BUSCOs (D)	31	1.9	25	1.5	29	1.8
Fragmented BUSCOs (F)	14	0.9	15	0.9	11	0.7
Missing BUSCOs (M)	35	2.2	32	2.0	22	1.3
Total BUSCO groups searched	1,614	100.0	1,614	100.0	1,614	100.0

Supplementary Table 8. Statistics of centromeric regions in Y476 genome.

Chromosome	Hap1				Hap2				Primary			
	CentO sequence location		Total no. of units	Total length (bp)	*CentO* sequence location		Total no. of units	Total length (bp)	*CentO* sequence location		Total no. of units	Total length (bp)
	Start	End			Start	End			Start	End		
Chr1	17,603,114	17,691,859	300	49,558	17,990,014	18,126,303	242	39,484	17,603,116	17,691,861	300	48,573
Chr2	14,097,045	14,191,372	474	77,693	13,817,586	13,911,913	474	77,669	14,097,054	14,191,381	474	77,912
Chr3	21,551,062	21,914,263	485	77,469	21,731,351	22,181,977	315	50,059	21,850,619	22,301,245	315	51,521
Chr4	10,989,229	11,155,366	306	48,839	10,569,577	11,230,187	583	97,568	10,989,231	11,155,368	306	50,508
Chr5	12,641,883	12,736,428	541	89,642	13,208,989	13,341,245	767	125,458	13,311,893	13,406,559	542	89,112
Chr6	17,034,882	17,074,919	43	6,793	17,877,973	17,987,850	520	82,817	17,877,962	17,987,839	520	84,665
Chr7	12,460,567	13,108,603	40	6,525	12,940,400	13,416,675	653	108,812	13,480,382	14,128,419	40	6,442
Chr8	14,014,749	14,938,597	263	42,165	14,009,830	14,107,856	492	79,549	14,088,067	14,180,111	255	41,861
Chr9	4,653,188	5,075,226	177	29,731	2,820,363	3,447,644	319	53,032	4,653,188	5,075,226	177	29,131
Chr10	10,047,300	10,048,286	7	1,024	10,073,518	10,074,503	7	1,031	10,048,134	10,049,120	7	1,072
Chr11	13,604,408	13,972,812	1,556	259,388	13,283,855	13,376,995	480	80,778	13,604,413	13,972,816	1,556	255,346
Chr12	12,284,304	12,777,600	805	134,098	12,332,728	13,134,096	1,604	266,529	12,351,528	12,844,824	805	133,499

Supplementary Table 9. General statistics of the telomeres for Y476 genome.

Chromosome	Hap1				Hap2				Primary			
	Left repeat number	Left repeat length (bp)	Right repeat number	Rigth repeat length (bp)	Left repeat number	Left repeat length (bp)	Right repeat number	Rigth repeat length (bp)	Left repeat number	Left repeat length (bp)	Right repeat number	Rigth repeat length (bp)
Chr1	1,289	8,880	854	5,759	1,452	9,963	1,825	12,633	1,329	9,060	1,870	12,847
Chr2	1,834	12,666	1,189	8,046	1,165	7,963	597	3,934	1,877	12,872	620	4,052
Chr3	705	4,670	1,364	9,278	1,013	6,838	1,367	9,032	727	4,783	1,393	9,157
Chr4	1,195	8,154	1,013	6,799	977	6,620	996	6,596	1,236	8,363	1,050	6,973
Chr5	1,676	11,350	892	6,127	1,164	7,944	1,129	7,666	1,176	7,964	918	6,255
Chr6	1,093	7,324	746	4,975	1,316	8,071	1,216	8,319	1,454	8,713	1,244	8,456
Chr7	1,230	8,407	930	6,352	1,059	7,029	660	4,457	1,100	7,275	953	6,467
Chr8	1,067	7,251	1,231	8,221	1,208	8,338	1,115	7,580	1,266	8,604	1,148	7,737
Chr9	\	\	878	5,636	\	\	834	5,654	\	\	920	5,843
Chr10	\	\	802	5,423	\	\	987	6,616	\	\	814	5,467
Chr11	971	6,601	1,354	8,857	919	6,192	898	6,226	1,001	6,757	904	6,262
Chr12	934	6,381	1,030	6,753	1,533	10,569	913	6,263	1,575	10,774	1,225	7,673

Supplementary Table 10. GO enrichment of specific gene families in Y476 compared to Nip and 9311 (A two-sided hypergeometric test was employed to analysis the GO enrichment, which were made for multiple comparisons by the Benjamini-Hochberg procedure to control the False Discovery Rate (FDR).).

GO ID	Number of gene families	Functional categories	Ontology	PValue
GO:0015074	5	DNA integration	biological_process	1.20E-09
GO:0044826	2	viral genome integration into host DNA	biological_process	1.62E-05
GO:0006811	3	ion transport	biological_process	0.000119532
GO:0003964	2	RNA-directed DNA polymerase activity	molecular_function	0.000160356
GO:0032197	2	transposition, RNA-mediated	biological_process	0.000334092
GO:0018298	2	protein-chromophore linkage	biological_process	0.000861251
GO:0010044	2	response to aluminum ion	biological_process	0.000861251
GO:0051539	2	4 iron, 4 sulfur cluster binding	molecular_function	0.001211774
GO:0006897	2	endocytosis	biological_process	0.002330327

Supplementary Table 11. Statistics of the top 10 Pfam and GO terms annotated by novel genes in Y476.

ID	gene number	Functional categories	Database
NB-ARC	106	Disease-resistant domain	Pfam
Px_N	78		Pfam
Pkinase	64		Pfam
PK_Tyr_Ser-THR	53	Protein tyrosine and serine/threonine kinase	Pfam
LRR_8	49	Disease-resistant domain	Pfam
PPR	36		Pfam
GUB_WAK_bind	34	Wall-associated receptor kinase galacturonan-binding	Pfam
LRRNT_2	33		Pfam
F-box	28		Pfam
PPR_2	27	PPR repeat family	Pfam
GO:0005515	179	protein binding	GO
GO:0005524	118	ATP binding	GO
GO:0004672	109	protein kinase activity	GO
GO:0006468	109	protein phosphorylation	GO
GO:0003531	103	voltage-gated potassium channel activity	GO
GO:0003676	84	nucleic acid binding	GO
GO:0055114	56	obsolete oxidation-reduction process	GO
GO:0003677	53	DNA binding	GO
GO:0008270	39	zinc ion binding	GO
GO:0006355	38	regulation of DNA-templated transcription	GO

Supplementary Table 12. Identification of tandem repeat genes in Y476.

Cluster ID	Annotation	Number of genes in Nip	Number of genes in 9311	Number of tandem repeat genes in Y476
OG0000050	NBS-LRR disease resistance	3	0	39
OG0000075	Regulate the expression of genes involved in rice grain development	1	3	32
OG0000116	Related to bacterial blight resistance	6	6	9
OG0000321	Related to rice blast resistance	4	4	9
OG0000326	Related to rice fertility	4	2	11
OG0000351	protein kinase domain containing protein	2	0	11
OG0000690	Resistance to rice brown planthopper	1	1	10
OG0003731	Related to creeping growth	1	0	6
OG0003702	Related to seedling biomass	2	0	5
OG0002402	disease resistance RPP13-like protein 1	1	1	6
OG0000697	dehydration stress-induced protein	2	2	8

Supplementary Table 13. Segment length distribution in the CSSL/9311 population.

Segment length (cM)	Segment number	Percentage (%)
0-5	630	43.54
5-10	308	21.29
10-15	164	11.33
15-20	104	7.19
20-30	104	7.19
＞30	137	9.47

Supplementary Table 14. Distribution of segments number in the CSSL/9311 population.

Segment length (cM)	Number of lines	Percentage (%)
0-5	116	58.59
6-10	52	26.26
11-20	16	8.08
21-30	7	3.54
31-40	5	2.53
＞40	2	1.01

Supplementary Table 15. Segments length distribution in the CSSL/Nip population.

Segment length (cM)	Segment number	Percentage (%)
0-5	1,791	26.10
5-10	1,513	22.05
10-15	1,199	17.47
15-20	584	8.51
20-30	1,032	15.04
＞30	744	10.84

Supplementary Table 16. Distribution of segments number in the CSSL/Nip population.

Segment length (cM)	Number of lines	Percentage (%)
0-10	45	20.0
11-20	44	19.6
21-30	19	8.4
31-40	31	13.8
41-50	48	21.3
＞50	38	16.9

Supplementary Table 17. Statistics of nine agronomic traits in Nip and CSSLs in the three environments.

Trait	Environment	Nip	Mean ± SD (CSSLs)	range (CSSLs)	CV(%) (CSSLs)
Plant height (cm)	2020Beijing	120.00	134.14 ± 26.27	69.00-207.20	19.59
Plant height (cm)	2021Hainan	73.13	110.05 ± 22.91	53.00-176.88	20.82
Plant height (cm)	2021Beijing	115.40	124.02 ± 23.45	60.25-197.10	18.91
panicle length (cm)	2020Beijing	19.64	23.34 ± 3.35	16.90-32.88	14.36
panicle length (cm)	2021Hainan	21.68	19.80 ± 2.98	13.60-29.93	15.07
panicle length (cm)	2021Beijing	20.76	23.20 ± 3.24	17.52-32.68	13.97
Number of tillers	2020Beijing	12.00	13.80 ± 4.97	5.20-36.50	36.00
Number of tillers	2021Hainan	17.00	14.72 ± 4.15	7.25-30.33	28.22
Number of tillers	2021Beijing	10.80	12.53 ± 4.50	4.80-36.5	35.89
Flag leaf length (cm)	2020Beijing	31.60	36.22 ± 6.81	19.97-60	18.80
Flag leaf length (cm)	2021Hainan	22.05	24.81 ± 4.76	13.8-41.05	19.19
Flag leaf length (cm)	2021Beijing	29.17	32.90 ± 5.41	19.83-53.1	16.43
Flag leaf width (cm)	2020Beijing	1.42	1.69 ± 0.33	1.02-2.75	19.56
Flag leaf width (cm)	2021Hainan	1.32	1.55 ± 0.27	0.94-2.45	17.71
Flag leaf width (cm)	2021Beijing	1.42	1.57 ± 0.32	0.96-2.80	20.40
Grain length (mm)	2020Beijing	6.99	7.94 ± 0.82	6.18-10.12	10.32
Grain length (mm)	2021Hainan	6.90	7.71 ± 0.75	6.04-9.52	9.71
Grain length (mm)	2021Beijing	6.98	8.01 ± 0.92	5.71-10.30	11.52
grain width (mm)	2020Beijing	3.18	2.90 ± 0.30	2.22-3.68	10.41
grain width (mm)	2021Hainan	3.22	2.85 ± 0.23	2.27-3.48	8.12
grain width (mm)	2021Beijing	3.24	2.96 ± 0.29	2.25-3.65	9.84
1000 grain weight (g）	2020Beijing	22.89	23 ± 3.54	13.8-31.22	15.39
1000 grain weight (g)	2021Hainan	24.26	22.81 ± 3.25	13.63-31.28	14.26
1000 grain weight (g)	2021Beijing	24.05	23.12 ± 3.66	12.58-32.02	15.83
Grain length width ratio	2020Beijing	2.20	2.79 ± 0.44	2.05-4.05	15.88
Grain length width ratio	2021Hainan	2.16	2.75 ± 0.40	1.97-3.90	14.75
Grain length width ratio	2021Beijing	2.17	2.75 ± 0.48	1.95-4.22	17.36

Supplementary Table 18. Three QTLs associated with salt tolerance were identified using CSSL/Nip population.

Trait	Peak marker	Flanking markers	LOD	PVE (%)	Add
Salt tolerance	S1_30170531	S1_30105830-S1_30184066	2.91	3.54	0.24
Salt tolerance	S2_4579633	S2_4576909-S2_4669781	12.26	15.31	-0.57
Salt tolerance	S2_8247382	S2_8246291-S2_8264241	4.23	5.00	0.35

Supplementary Table 19. List of four genes in the salt tolerance QTL locus S2_4579633.

Chromosome	Start	End	Gene ID	Annotation
Chr2	4,594,375	4,595,386	*LOC_Os02g08510*	ZOS2-04 - C2H2 zinc finger protein expressed
Chr2	4,599,038	4,601,200	*LOC_Os02g08520*	peptidase T1 family putative expressed
Chr2	4,604,012	4,607,666	*LOC_Os02g08530*	protein kinase family protein putative expressed
Chr2	4,616,830	4,622,111	*LOC_Os02g08540*	expressed protein

Supplementary Table 20. Variations in the CDS region of LOC_Os02g08540 between N133 and Nip.

Chromosome	Genomic location	Nip	CSSL
Chr2	4617314	A	G
Chr2	4619336	C	G
Chr2	4621692	G	A

Supplementary Table 21. QTLs associated with rice blast resistance were identified using the CSSL/Nip population.

Trait	Peak marker	Flanking markers	LOD	PVE (%)	Add
rice blast resistance	S1_39069938	S1_39058511-S1_39074251	8.43	9.60	-0.54
rice blast resistance	S2_6398734	S2_6145849-S2_6479789	7.17	7.79	-1.41
rice blast resistance	S3_27819424	S3_27776849-S3_27894144	2.99	3.37	-0.27
rice blast resistance	S7_21365207	S7_21349910-S7_21380932	10.93	12.12	-1.76
rice blast resistance	S8_3513954	S8_3392938-S8_3548822	6.35	6.86	-0.46
rice blast resistance	S8_5625460	S8_5600848-S8_5751765	4.72	5.34	-0.37
rice blast resistance	S11_23867299	S11_23860806-S11_23938332	4.99	5.48	-0.39

Supplementary Table 22. List of genes in rice blast resistance QTL locus *S7_21365207*.

Chromosome	Start	End	Gene ID	Annotation
Chr7	21,359,653	21,366,555	*LOC_Os07g35660*	TKL_IRAK_DUF26-lc.21 - DUF26 kinases have homology to DUF26 containing loci expressed
Chr7	21,367,512	21,367,919	*LOC_Os07g35670*	hypothetical protein
Chr7	21,367,969	21,374,694	*LOC_Os07g35680*	TKL_IRAK_DUF26-lc22 - DUF26 kinases have homology to DUF26 containing loci expressed

Supplementary Table 23. Three environments in which the CSSL/Nip population were investigated.

Environment	Replication	Crop location	Cropping season	Sowing date	Transplanting date
E1	3	Shunyi, Beijing N40.20°, E115.51°	May-Oct 2020	5-May-20	1-Jun-20
E2	3	Sanya, Hainan N18.15°, E109.3°	Dec 2020-May 2021	5-Dec-20	5-Jan-21
E3	3	Shunyi, Beijing N40.20°, E115.51°	May-Oct 2021	5-May-21	1-Jun-21

83. Tao, Y. et al. *RGG1*, Involved in the cytokinin regulatory pathway, controls grain size in rice. *Rice* **13**, 76 (2020).
84. Zhao, M. et al. Regulation of *OsmiR156h* through alternative polyadenylation improves grain yield in rice. *PloS one* **10**, e0126154 (2015).
85. Kurotani, K. I. et al. Overexpression of a CYP94 family gene *CYP94C2b* increases internode length and plant height in rice. *Plant Signal. Behav.* **10**, e1046667 (2015).
86. Tang, L. et al. *OsABF1* represses gibberellin biosynthesis to regulate plant height and seed germination in rice (*Oryza sativa* L.). *Int. J. Mol. Sci*. **22**, 12220 (2021).
87. Zhang, Y. et al. Gibberellin homeostasis and plant height control by *EUI* and a role for gibberellin in root gravity responses in rice. *Cell Res*. **18**, 412-421 (2008)
88. Ding, C. et al. Transcription factor *OsbZIP49* controls tiller angle and plant architecture through the induction of indole-3-acetic acid-amido synthetases in rice. *Plant J.* **108**, 1346-1364 (2021).
89. Chen, S. et al. *OsRRM*, a Spen-like rice gene expressed specifically in the endosperm. *Cell Res*. **17**, 713-721 (2007).
90. Jang, S. et al. Rice leaf angle and grain size are affected by the OsBUL1 transcriptional activator complex. *Plant Physiol*. **173**, 688-702 (2017).
91. Xiao, Y. et al. Endoplasmic reticulum-localized PURINE PERMEASE1 regulates plant height and grain weight by modulating cytokinin distribution in rice. *Front Plant Sci*. **11**, 618560 (2020).
92. Liu, E. et al. Identification of a candidate gene for panicle length in rice (*Oryza sativa* L.) via association and linkage analysis. *Front Plant Sci*. **7**, 596 (2016).
93. Shao, G. et al. Tiller bud formation regulators *MOC1* and *MOC3* cooperatively promote tiller bud outgrowth by activating *FON1* expression in rice. *Mol. Plant* **12**, 1090-1102 (2019).
94. Yeh, S. et al. 2015. Down-regulation of cytokinin oxidase 2 expression increases tiller number and improves rice yield. *Rice* **8**, 36 (2015).
95. Xiong, G. et al. *Leafy head2*, which encodes a putative RNA-binding protein, regulates shoot development of rice. *Cell Res*. **16**, 267-276 (2006).
96. Zhang, Z. et al. The 14-3-3 protein GF14f negatively affects grain filling of inferior spikelets of rice (*Oryza sativa* L.). *Plant J.* **99**, 344-358 (2019).
97. Li, Y. et al. Natural variation in *GS5* plays an important role in regulating grain size and yield

in rice. *Nat. Genet*. **43**, 1266-1269 (2011).

98. Wan, X. et al. Quantitative trait loci (QTL) analysis for rice grain width and fine mapping of an identified QTL allele *gw-5* in a recombination hotspot region on chromosome 5. *Genetics* **179**, 2239-2252 (2008).

99. Park, J. R., Resolus, D. & Kim, K. M. *OsBRKq1*, related grain size mapping, and identification of grain shape based on qtl mapping in rice. *Int. J. Mol. Sci*. **22**, 2289 (2021).

100. Yoon, D. H. et al. Overexpression of *OsCYP19-4* increases tolerance to cold stress and enhances grain yield in rice (*Oryza sativa*). *J. Exp. Bot*. **67**, 69-82 (2016).
