# Supplementary material for: Haplotype-resolved gapless genome and chromosome segment substitution lines facilitate gene identification in wild rice
Source: Nat Commun. 2024 May 29;15:4573. doi: 10.1038/s41467-024-48845-6 (PMC11137157; doi:10.1038/s41467-024-48845-6)
Supplement: Supplementary file 3 — Description of Additional Supplementary Files [file 41467_2024_48845_MOESM3_ESM.pdf]

Description of Additional Supplementary Files

File Name: Supplementary Data 1

Description: Evaluating the quality of genomes based on VerityMap and T2T-polish.

File Name: Supplementary Data 2

Description: SVs statistics between Hap1/Hap2/Primary genomes and Nip.

File Name: Supplementary Data 3

Description: The read coverage depth of copy genes in two gene clusters.

File Name: Supplementary Data 4

Description: QTL mapping of nine agronomic traits using the CSSL/Nip population.

File Name: Supplementary Data 5

Description: SVs Statistics on the QTLs.

File Name: Supplementary Data 6

Description: Variations in the CDS region of *LOC_Os07g35680* between N154 and Nip.

File Name: Supplementary Data 7

Description: Primers used in the study.
