# Supplementary material for: Haplotype-resolved gapless genome and chromosome segment substitution lines facilitate gene identification in wild rice
Source: Nat Commun. 2024 May 29;15:4573. doi: 10.1038/s41467-024-48845-6 (PMC11137157; doi:10.1038/s41467-024-48845-6)
Supplement: Supplementary file 11 — Reporting Summary [file 41467_2024_48845_MOESM11_ESM.pdf]

nature portfolio

Corresponding author(s): NCOMMS-23-06511

Last updated by author(s): Apr 27, 2024

Reporting Summary

Nature Portfolio wishes to improve the reproducibility of the work that we publish. This form provides structure for consistency and transparency in reporting. For further information on Nature Portfolio policies, see our Editorial Policies and the Editorial Policy Checklist.

Statistics

For all statistical analyses, confirm that the following items are present in the figure legend, table legend, main text, or Methods section.

n/a	Confirmed	
☐	☒	The exact sample size (*n*) for each experimental group/condition, given as a discrete number and unit of measurement
☐	☒	A statement on whether measurements were taken from distinct samples or whether the same sample was measured repeatedly
☐	☒	The statistical test(s) used AND whether they are one- or two-sided<br>*Only common tests should be described solely by name; describe more complex techniques in the Methods section.*
☒	☐	A description of all covariates tested
☐	☒	A description of any assumptions or corrections, such as tests of normality and adjustment for multiple comparisons
☐	☒	A full description of the statistical parameters including central tendency (e.g. means) or other basic estimates (e.g. regression coefficient) AND variation (e.g. standard deviation) or associated estimates of uncertainty (e.g. confidence intervals)
☐	☒	For null hypothesis testing, the test statistic (e.g. *F*, *t*, *r*) with confidence intervals, effect sizes, degrees of freedom and *P* value noted<br>*Give P values as exact values whenever suitable.*
☒	☐	For Bayesian analysis, information on the choice of priors and Markov chain Monte Carlo settings
☒	☐	For hierarchical and complex designs, identification of the appropriate level for tests and full reporting of outcomes
☒	☐	Estimates of effect sizes (e.g. Cohen's *d*, Pearson's *r*), indicating how they were calculated

Our web collection on statistics for biologists contains articles on many of the points above.

Software and code

Policy information about availability of computer code

Data collection	No software was used for data collection.
Data analysis	Jellyfish (version 2.3.0), GenomeScope (version 1.0), Hifiasm (version 0.16.1), NextDenovo (version 2.5.0), HiC-Pro (v2.11.1), 3D-DNA (version 180922), RagTag (version 2.1.0), HiCPlotter (version 0.6.6), EDTA(version 2.0.0), BUSCO (version 5.4.3), Racon (version 1.4.20), Merfin (version 1.0), Qualimap2 (version 2.2.2), Merqury (version 1.3), HMMER (version 3.2), RepeatMasker (version 4.1.1), Augustus (version 3.2.3), GlimmerHMM (version 3.0.4), Braker2 (version 2.1.6), Exonerate (version 2.2.0), EVidenceModeler (version 1.1.1), Blast (version 2.10.0+), InterProScan (version 5.24-63.0), tRNAscan-SE (version 1.3.1), RNAmmer (version 1.2), MUMmer (version 4.0.0rc1), MUMandCo (version 2.4.2), Syri (version 1.6), QTL IciMapping (v4.2), SPSS, ADMIXTURE (version 1.3.0), Orthofinder (version 2.5.4), Stringtie (version1.2.3), TransDecoder (version 2.0), SamTools (version0.1.19), BWA(version 0.7.17),GATK4, Hisat2(version 2.2.1), VerityMap, T2T-Polish, minimap2, Integrative Genomics Viewer, Merqury QV

For manuscripts utilizing custom algorithms or software that are central to the research but not yet described in published literature, software must be made available to editors and reviewers. We strongly encourage code deposition in a community repository (e.g. GitHub). See the Nature Portfolio guidelines for submitting code & software for further information.

Data

Policy information about availability of data

All manuscripts must include a data availability statement. This statement should provide the following information, where applicable:

- Accession codes, unique identifiers, or web links for publicly available datasets
- A description of any restrictions on data availability
- For clinical datasets or third party data, please ensure that the statement adheres to our policy

the raw sequencing data and genome assembly in this study are available in the National Center for Biotechnology Information (NCBI) and National Genomics Data Center (NGDC), and the accession numbers are PRJNA1029807 (NCBI) and PRJCA015108 (NGDC: https://ngdc.cncb.ac.cn/bioproject/browse/PRJCA015108). The genome assembly and annotation files are available at the website https://figshare.com/articles/dataset/Genome_sequence_and_annotation_of_Hap1_Hap2_and_primary_of_Y476/24798696.

Human research participants

Policy information about studies involving human research participants and Sex and Gender in Research.

Reporting on sex and gender	Not suitable for our study.
Population characteristics	Not suitable for our study.
Recruitment	Not suitable for our study.
Ethics oversight	Not suitable for our study.

Note that full information on the approval of the study protocol must also be provided in the manuscript.

Field-specific reporting

Please select the one below that is the best fit for your research. If you are not sure, read the appropriate sections before making your selection.

☒ Life sciences ☐ Behavioural & social sciences ☐ Ecological, evolutionary & environmental sciences

For a reference copy of the document with all sections, see nature.com/documents/nr-reporting-summary-flat.pdf

Life sciences study design

All studies must disclose on these points even when the disclosure is negative.

Sample size	The species used for PacBio sequencing, Nanopore Sequencing and Bionano Sequencing is Oryza rufipogon (sample size, n=1). The CSSL/Nip (sample size, n=225) covers the whole genome of wild rice.
Data exclusions	No data were excluded from analysis.
Replication	All experiments have been repeated successfully at least three times.
Randomization	For Phenotype data statistics, the measured samples were randomly selected. For RNA-seq, the samples were randomly selected.
Blinding	Blinding was not relevant for this study.

Reporting for specific materials, systems and methods

We require information from authors about some types of materials, experimental systems and methods used in many studies. Here, indicate whether each material, system or method listed is relevant to your study. If you are not sure if a list item applies to your research, read the appropriate section before selecting a response.

Materials & experimental systems

n/a	Involved in the study	
☒	☐	Antibodies
☒	☐	Eukaryotic cell lines
☒	☐	Palaeontology and archaeology
☒	☐	Animals and other organisms
☒	☐	Clinical data
☒	☐	Dual use research of concern

Methods

n/a	Involved in the study	
☒	☐	ChIP-seq
☒	☐	Flow cytometry
☒	☐	MRI-based neuroimaging
